# Accre 8 emerging point of care CLIA system for vitamin B12 assessment compared with three established assays

**DOI:** 10.1038/s41598-025-97503-4

**Published:** 2025-04-17

**Authors:** Farah M. Trad, Tasneem AlHamad, Nadin Younes, Shaden Abunasser, Salma Younes, Parveen B. Nizamuddin, Dayana El Chaar, Israa M. Salameh, Nader I. Al-dewik, Wanida Laiwattanapaisal, Pattramon Aungbamnet, Pollanat Loungjinda, Palanee Ammaranond, Meng Li, Laith J. Abu-Raddad, Gheyath K. Nasrallah

**Affiliations:** 1 Biomedical Research Center, QU Health, Qatar University, P.O. Box 2713, Doha, Qatar; 2 Biomedical Sciences Department, College of Health Sciences, QU Health, Qatar University, P.O. Box 2713, Doha, Qatar; 3 Faculty of Medicine, Department of Nutritional Sciences, University of Toronto, 27 King’s College Cir, Toronto, ON M5S 1 A1 Canada; 4 Department of Research, Women’s Wellness and Research Center, Hamad Medical Corporation, P.O.BOX. 3050, Doha, Qatar; 5 Department of Clinical Chemistry, Faculty of Allied Health Sciences, Chulalongkorn University, Patumwan, Bangkok, 10330 Thailand; 6 Medical Technology Unit, Health Sciences Service Center, Faculty of Allied Health Sciences, Chulalongkorn University, Bangkok, Thailand; 7 Department of transfusion medicine and clinical microbiology, Faculty of Allied Health Sciences, Chulalongkorn University, Bangkok, 10330 Thailand; 8 Guangzhou Wondfo Biotech Co., Ltd, No. 8 Lizhishan Road, Science City, Huangpu District, Guangzhou, 510663 China; 9 Weill Cornell Medical College – Qatar, Cornell University, Qatar Foundation - Education City, Doha, Qatar

**Keywords:** Vitamin B12, Deficiency, Immunoassay, Accre 8, CLIA, Point-of-Care, Biotechnology, Assay systems

## Abstract

Accurate Vitamin B12 (Vit B12) quantification is essential for diagnosing deficiencies linked to neurological and hematological disorders. The Accre 8 Point-of-Care (POC) Chemiluminescent Immunoassay (CLIA) system offers a compact design, rapid single-step operation, and minimal calibration requirements. This study evaluates Accre 8’s performance against established CLIA immunoassays (Abbott and Roche) and LC-MS/MS, the gold standard for Vit B12 quantification. A total of 297 serum samples, spanning deficient to sufficient Vit B12 levels, were analyzed. Accre 8 demonstrated a strong correlation with LC-MS/MS (*r* = 0.94, *p* < 0.001), with median Vit B12 levels closely aligning with LC-MS/MS (256.0 pmol/L). Accre 8 exhibited high sensitivity (96.9%) and specificity (86.7%), with Cohen’s Kappa agreement (0.76). Bland-Altman analysis showed a mean bias of - 18.5%, while Passing-Bablok regression indicated proportional bias at higher concentrations (slope = 1.44). ROC analysis confirmed excellent diagnostic accuracy (AUC = 0.98). Accre 8’s strong diagnostic performance, minimal calibration needs, and low sample volume requirements position it as a practical alternative to conventional CLIA systems for Vit B12 assessment, particularly in clinical and resource-limited settings. These findings support its potential integration into routine diagnostic workflows for Vit B12 deficiency screening and monitoring.

**Supplementary Information:**

The online version contains supplementary material available at 10.1038/s41598-025-97503-4.

## Introduction

Vit B12, also known as cyanocobalamin, is a water-soluble vitamin essential for physiological processes, including DNA synthesis, red blood cell production, and maintaining nervous system health^1,2^. It is primarily obtained from animal-derived foods, such as meat, liver, fish, eggs, and dairy products^1,3,4^. Its significance is particularly critical for vulnerable populations, including vegans, pregnant women, and infants^1^. Such at-risk populations can suffer from deficiencies in Vit B12, leading to significant health issues such as anemia, developmental delays, and neurological complications such as cognitive impairment^2,5^. Accurate diagnosis of Vit B12 deficiency is crucial for the timely diagnosis and treatment^6^.

Vit B12 deficiency is commonly diagnosed by measuring serum B12 levels, especially in high-risk populations^7^. Pregnant women with Vit B12 deficiency face an increased risk of hyperglycemia, insulin resistance, obesity, and dyslipidemia, potentially compromising both maternal health and fetal development^8,9^. Additionally, newborn screening can detect Vit B12 deficiency, preventing neurological symptoms and reducing the likelihood of recurrence in subsequent pregnancies by facilitating treatment for both the mother and child^10^.

One of the key challenges in diagnosing Vit B12 deficiency is determining the appropriate cut-off values^11^. National studies from various countries have established different serum B12 cut-off values for defining deficiency (< 148 pmol/L), insufficiency (148–221 pmol/L), and sufficiency (> 256 pmol/L), with common thresholds set at < 148, < 200, and < 256 pmol/L, respectively^12^. This variability highlights the need for more reliable and standardized assays to improve diagnostic accuracy and ensure consistent clinical decision-making, particularly for at-risk populations^7^.

Chemiluminescent immunoassays (CLIAs) are widely employed in laboratories due to their high sensitivity, specificity, and suitability for high-throughput automation^13–15^. They have also been proven to be reliable and accurate for the diagnosis and monitoring of Vit B12^16^. Nonetheless, the variation among manufacturers of CLIA assays necessitates ongoing evaluation of their performance^17,18^, as these differences can significantly impact patients’ diagnoses and treatments^19^. Despite their advantages, CLIAs are not without limitations. Issues such as cross-reactivity and interference from heterophile antibodies can compromise accuracy, leading to false positives and misdiagnoses^20,21^. Addressing these limitations requires evaluating new platforms capable of providing consistent and accurate results across various clinical settings.

The Accre 8 CLIA POC system, developed by Wondfo Biotech Co., Ltd. and Tisenc Medical Devices Co., Ltd. represents a significant advancement in point-of-care diagnostics, offering several unique advantages. Its compact design eliminates the need for large workspaces, while the streamlined single-step process removes the requirement for washing solutions, reducing operational complexity. The system’s minimal sample volume requirement makes it particularly suitable for applications such as neonatal screening, and the reduced need for frequent calibration ensures consistent performance with minimal maintenance, making it ideal for small laboratories and resource-limited settings.

Despite these advantages, the Accre 8 CLIA POC system has not been validated in the literature for any diagnostic parameter, including Vit B12. To address this gap, we evaluated the performance of Accre 8 along with established CLIA immunoassays including Abbott and Roche against LC-MS/MS, which is widely recognized as the gold standard for Vit B12 quantification^22–29^. This study offers the first in-depth performance analysis of the Accre 8 system, highlighting its potential as a practical and reliable diagnostic tool for clinical applications.

## Materials and methods

### Study design and clinical samples

The performance and validation of the Accre 8 – Automatic Chemiluminescence Immunoassay Analyzer (Wondfo Biotech Co., Ltd. and Tisenc Medical Devices Co., Ltd., China) (Figure S1) were evaluated by comparing it with LC-MS/MS, the reference method used in this study. Additionally, it was also assessed against two FDA-approved CLIA fully automated analyzers: Roche Cobas 6000e (Roche Diagnostics GmbH, Mannheim, Germany) and Architect ci4100 analyzer (Abbott Laboratories, Abbott Park, IL, USA).

A total of 297 serum samples were obtained from Qatar Biobank (QBB) for analysis using CLIA analyzers. All serum samples were aliquoted and stored at − 80 °C to prevent degradation from freeze-thaw cycles. They were carefully selected to represent a wide range of Vit B12 concentrations, including deficient, normal, and sufficient levels. The assays were conducted in different laboratory locations at different times due to logistical constraints and equipment availability. Roche assay measurements had already been performed at QBB, while Abbott and LC-MS/MS assays were conducted in separate laboratories, and Accre 8 measurements were performed in our lab. Since the assays were not run simultaneously or in the same location, several pre-analytical and analytical measures were implemented to ensure comparability and minimize batch effects. To maintain consistency, each assay was performed using a single lot number (batch no.), and strict adherence to manufacturer protocols and calibration procedures was ensured for all instruments. The Roche, Abbott, and LC-MS/MS assays were conducted in an external International Organization for Standardization (ISO) and College of American Pathologists (CAP) - accredited reference laboratory, which adheres to stringent quality control protocols based on international standards.

Among the tested samples, 129 individuals were identified as having sufficient Vit B12 levels, while 168 were classified as deficient based on Roche sample measurements. The classification used the WHO-defined cutoff of < 221 pmol/L, a threshold commonly applied in the Middle East^30,31^. The manufacturer-provided cutoff values were not used, as they were not representative of our study population.

**Fig. 1:**
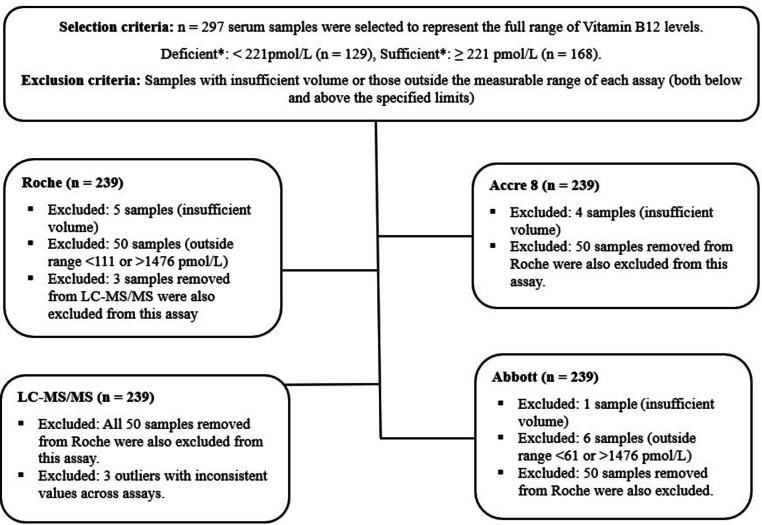
**Flowchart illustrating the inclusion and exclusion criteria for serum samples analyzed using Roche**,** Abbott**,** Accre 8**,** and LC-MS/MS.** A total of 297 serum samples were initially selected to represent a full range of Vit B12 levels, categorized as deficient (< 221 pmol/L, *n* = 97) or sufficient (≥ 221 pmol/L, *n* = 142). Samples were excluded due to insufficient volume or Vit B12 concentrations outside the measurable range of each assay: < 111 pmol/L or > 1476 pmol/L for Roche, < 61 pmol/L or > 1476 pmol/L for Abbott. Additionally, 50 samples removed from Roche were also excluded from Accre 8 and Abbott assays. Three samples were excluded from LC-MS/MS due to inconsistencies with other assays. Excluded samples were omitted from quantitative assay analyses but retained for diagnostic performance assessments, including sensitivity, specificity, and agreement evaluations. The Vit B12 deficiency threshold was based on the World Health Organization (WHO) cut-off value.

## CLIA diagnostic assay methodologies

### Accre 8 analyzer

The Accre 8 Analyzer (Ref. W01G, Tisenc Medical Devices Co., Ltd., China, Acquired by Wondfo Biotech Co., Ltd., China) Vit B12 assay employs a competitive immunoassay principle combined with enzyme catalytic chemiluminescence detection (ECLIA)^32^. The sample is manually added to a reagent cartridge containing pre-dispensed, ready-to-use reagents. Pre-treatment solutions 1 and 2 are mixed with the sample to release Vit B12 from its binding proteins during incubation at 37 °C. The sample then interacts with an alkaline phosphatase (ALP)-labeled swine intrinsic factor, forming an antibody-antigen complex. Magnetic particles labeled with intrinsic factor bind to the Vit B12 -ALP complexes during a second incubation step. The unbound substances are washed away using a magnetic field. A chemiluminescent substrate (APS- 5) is then added to produce a light-emitting reaction. The luminescence is measured by a photomultiplier tube (PMT) as relative light units (RLUs), with the Vit B12 concentration inversely proportional to the light intensity. Results are automatically calculated by the ACCER analyzer using a stored calibration curve, ensuring precise and efficient analysis. Detailed images illustrating the workflow and operational components of the Accre 8 CLIA POC system can be found in the supplementary materials (Figures S1A-S1 C).

## Analytical precision assessment

To evaluate the reproducibility of the Accre 8 assay, intra-assay (within-run) and inter-assay (between-run) precision were assessed using control samples at two concentration levels: low (~ 338.9 pmol/L) and high (~ 1492.3 pmol/L). Precision testing was performed following the Clinical and Laboratory Standards Institute (CLSI) guidelines, and results were reported as the coefficient of variation (CV%).

For intra-assay precision, five replicate measurements of each control level were analyzed within a single run. The CV% was 8.72% for low concentrations and 2.92% for high concentrations, indicating lower precision in the low range. For inter-assay precision, control samples were tested over five consecutive days, with five independent measurements per day. The overall CV% for inter-assay precision was 1.87% at low concentrations and 2.72% at high concentrations, demonstrating high reproducibility. The data for the analytical precision assessment, including inter- and intra-assay precision results, are provided in Table S1A and S1B in the Supplementary Material for reference (Table S1A-B).

The Roche, Abbott, and LC-MS/MS assays were conducted in an external ISO and CAP-accredited reference laboratory, which adheres to stringent quality control protocols based on international standards. As these assays were performed in different laboratories, precision data for these methods were not provided.

## Roche Cobas 6000e module analyzer

Roche analyzer (Ref No. 07212780, Roche Diagnostics GmbH, Germany) is the assay used in Hamad Medical Corporation (HMC) for the quantification of Vit B12^33^. This electrochemiluminescence immunoassay (ECLIA) uses a competitive binding principle with an intrinsic factor specific to Vit B12 and is completed in approximately 27 min. In the process, Vit B12 from the sample competes with biotin-labeled B12 for binding sites on a ruthenium-labeled intrinsic factor complex. This complex is captured on a solid phase via biotin-streptavidin interaction and microparticles are magnetically adhered to the electrode after unbound substances are washed away. Chemiluminescence is then measured by a photomultiplier to determine the Vit B12 concentrations using a two-point calibration.

### Architect ci4100 analyzer

The Abbott B12 assay (Ref No. 7 K61 - 25, Abbott Laboratories, USA) is a two-step process with automated sample pretreatment, designed to measure B12 levels in human serum and plasma samples using Chemiluminescent Microparticle Immunoassay (CMIA) technology with flexible Chemiflex assay protocols^34^. The sample is combined with Pre-Treatment Reagents 1, 2, and 3, then transferred into a Reaction Vessel (RV), where it mixes with assay diluent and intrinsic factor-coated paramagnetic microparticles, allowing B12 binding. After washing, a B12 acridinium-labeled conjugate is added forming a reaction mixture. Following another washing step, Pre-Trigger and Trigger Solutions initiate a chemiluminescent reaction. Relative light units (RLUs) are measured, inversely proportional to the B12 concentration in the sample.

## LC-MS/MS

Vit B12 analysis was conducted using an LC-MS/MS system (Shimadzu LCMS- 8030, Kyoto, Japan) equipped with a triple quadrupole mass spectrometer and atmospheric pressure chemical ionization (APCI) source, following modified protocols^25–27^. Serum samples (100 µL) were mixed with 300 µL HPLC-grade acetonitrile (Thermo-Fisher Scientific, Catalog No. 022927.K2, Waltham, MA, USA) to precipitate proteins, vortexed for 30 s, and centrifuged at 12,000 × g for 10 min. The supernatant, spiked with Vit B12-d5 as an internal standard (Sigma-Aldrich), was injected (10 µL) into a Shim-pack Scepter C18 - 300 column (2.1 × 150 mm, 3 μm) maintained at 25 °C. Chromatographic separation was achieved using gradient elution with acetonitrile (Phase A) and water containing 0.1% formic acid (Phase B) at a flow rate of 0.3 mL/min over 11 min. Positive-mode APCI was employed with a capillary voltage of 3.5 kV, source temperature of 105 °C, and desolvation temperature of 180 °C. Quantification was based on multiple reaction monitoring (MRM) transitions for Vit B12 (m/z 678.3 → 359.2) and its internal standard (m/z 683.3 → 364.2). Calibration covered a range of 10–1000 pg/mL, and the method was validated according to FDA guidelines for accuracy, precision, and recovery.

### Statistical analysis

The statistical analysis began by comparing the correlation between the data obtained from the three chemiluminescent assays (Accre 8, Roche and Abbott) and LC-MS/MS (the reference method). Additionally, Spearman’s correlation was also performed for Accre 8 with Abbott and Roche to further assess its relationship with the widely used CLIA assays. To maintain the integrity of method comparison and avoid bias in correlation analysis, these LC-MS/MS samples with inconsistent measurements based on deviations from other assay results were removed from the dataset before statistical evaluation. Differences between median values across assays were evaluated using the Kruskal-Wallis test, followed by Dunn’s multiple comparison test, with significance levels denoted as *p < 0.05, **p < 0.01, and ***p < 0.0001. As the data did not follow a normal distribution, median (M) and interquartile range (IQR) were used as the primary statistical descriptors, while mean (x̄) values were also included for reference and displayed on top of the graph. For two-group comparisons, statistical significance was assessed using a t-test. The Spearman correlation coefficient (r) was estimated to assess the monotonic relationship between the two variables, as the data were not normally distributed. The correlation strength was categorized as the following: *r* = 0–0.19 indicates a very weak or no correlation, *r* = 0.20–0.39 shows a weak correlation, *r* = 0.40–0.59 represents a moderate correlation, *r* = 0.60–0.79 shows a strong correlation, and *r*= 0.80–1.0 indicates a very strong correlation^35-38^.

Agreement between the CLIA methods and LC-MS/MS was assessed using Bland-Altman plots, which quantify bias by calculating limits of agreement (LOA). This graphical approach allowed for the assessment of systematic differences and trends in measurement discrepancies by determining the mean bias, 95% LOA, and the spread of differences across the measurement range^39,40^. Additionally, Passing-Bablok regression analysis was performed to detect systematic and proportional bias between methods. This non-parametric regression method is particularly suited for method comparison as it does not assume normality or constant variance. The regression equation, including confidence intervals for the intercept and slope, was computed to determine whether systematic or proportional bias was present. All Passing-Bablok regression analyses were conducted using MedCalc software (MedCalc Software Ltd, Ostend, Belgium)^41–43^.

Furthermore, concordance analysis was performed for Accre 8, Roche, Abbott and LC-MS/MS which included assessments of sensitivity, specificity, PPV, NPV, and Cohen’s Kappa Coefficient. Cohen’s Kappa values were used to indicate levels of agreement, with ≤ 0 showing no agreement, 0.01–0.20 as slight agreement, 0.21–0.40 as fair agreement, 0.41–0.60 as moderate agreement, 0.61–0.74 as good agreement, 0.75–0.80 as substantial agreement, and 0.81–1.00 almost perfect agreement^36,44–46^.

A Chi-Square test was also performed in GraphPad Prism to understand the association between the assays and used to calculate the confidence interval in GraphPad Prism. All statistical analyses, including the ROC curve and Bland-Altman plots, were conducted using GraphPad Prism (Version 10.3.1, GraphPad Software, Inc., San Diego, CA, USA).

## Results

### Spearman’s correlation reveals strong associations between assays

**Fig. 2:**
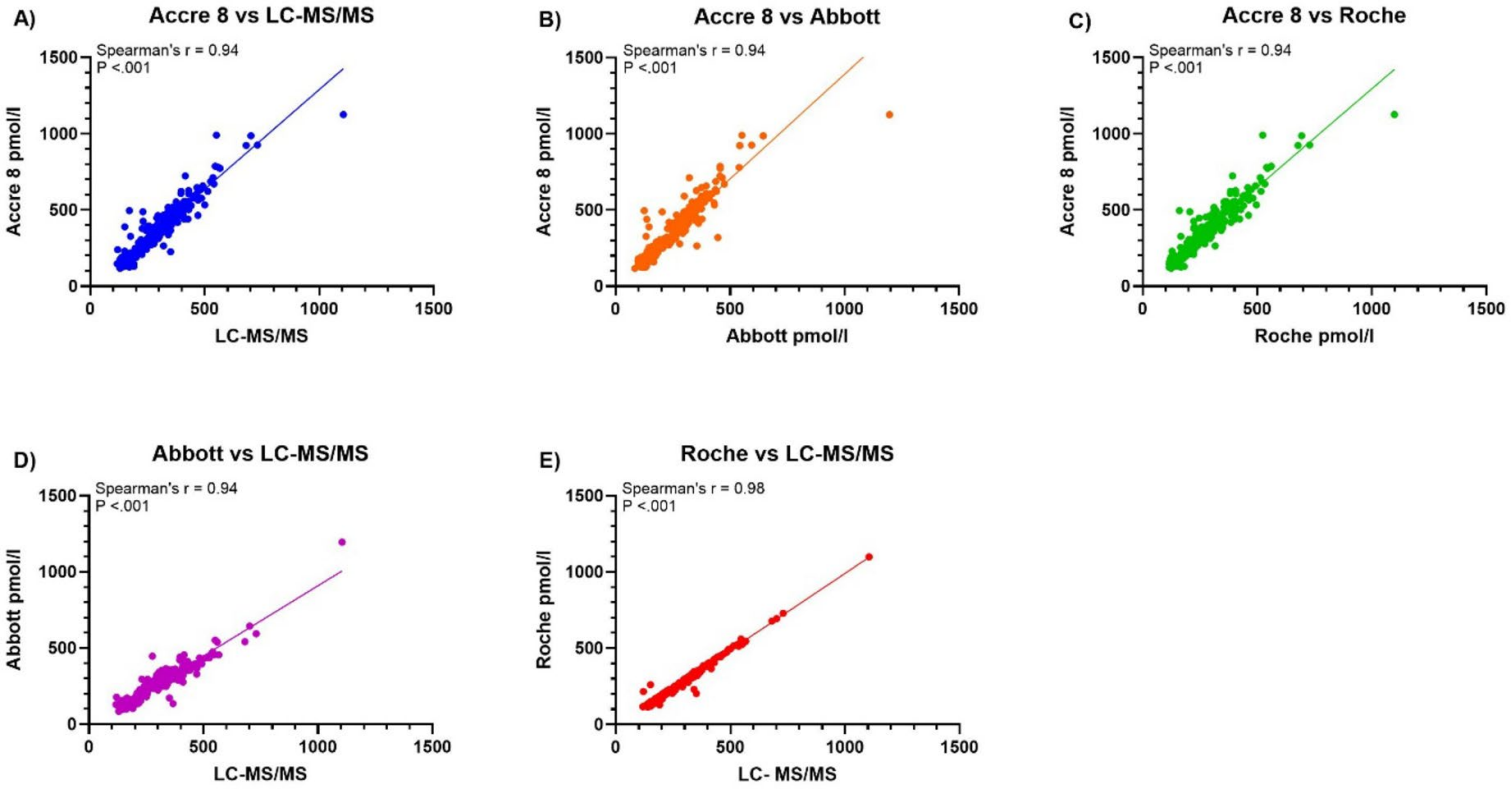
Correlation Analysis of Vit B12 levels between different assays. Figure 2 illustrates the correlation analysis of Vit B12 levels between different assays, assessing the strength of the relationship between LC-MS/MS and three CLIA-based methods using Spearman’s rank correlation coefficients (r). Plot A presents the correlation between Accre 8 and LC-MS/MS (*r* = 0.94, *p* < 0.001), while Plot B shows the correlation between Accre 8 and Roche (*r* = 0.94, *p* < 0.001). Plot C displays the correlation between Accre 8 and Abbott (*r* = 0.94, *p* < 0.001). Plot D illustrates the correlation between Abbott and LC-MS/MS (*r* = 0.94, *p* < 0.001), whereas Plot E presents the strongest correlation between Roche and LC-MS/MS (*r* = 0.98, *p* < 0.001). Each plot includes a diagonal reference line (y = x) for visual assessment of agreement.

Correlation analyses between LC-MS/MS and the three CLIA assays (Accre 8, Abbott, and Roche) were conducted to evaluate the consistency of Vit B12 quantification (Fig. 2A–E). The results demonstrated strong positive correlations across all comparisons, with Spearman’s correlation coefficients (r) ranging from 0.94 to 0.98 (*p* < 0.001 for all comparisons), indicating a high degree of agreement in Vit B12 measurements. In the comparison between Accre 8 and LC-MS/MS (Fig. 2A), the correlation coefficient was *r* = 0.94, showing a strong relationship between the two methods. Similarly, Abbott vs. LC-MS/MS (Fig. 2D) also exhibited a high correlation (*r* = 0.94), suggesting strong agreement with the reference method. Roche vs. LC-MS/MS (Fig. 2E) demonstrated the highest correlation (*r* = 0.98), indicating the closest alignment with LC-MS/MS. Additionally, correlation analyses were performed to assess the agreement between the three CLIA-based assays. The comparison between Accre 8 and Roche (Fig. 2B) showed a strong correlation (*r* = 0.94), as did Accre 8 vs. Abbott (Fig. 2C), further supporting the reliability of these assays for Vit B12 quantification. These findings highlight the strong agreement among the three immunoassays while reinforcing Roche’s close alignment with LC-MS/MS.

### Comparative distribution of Vit B12 levels across assays

**Fig. 3:**
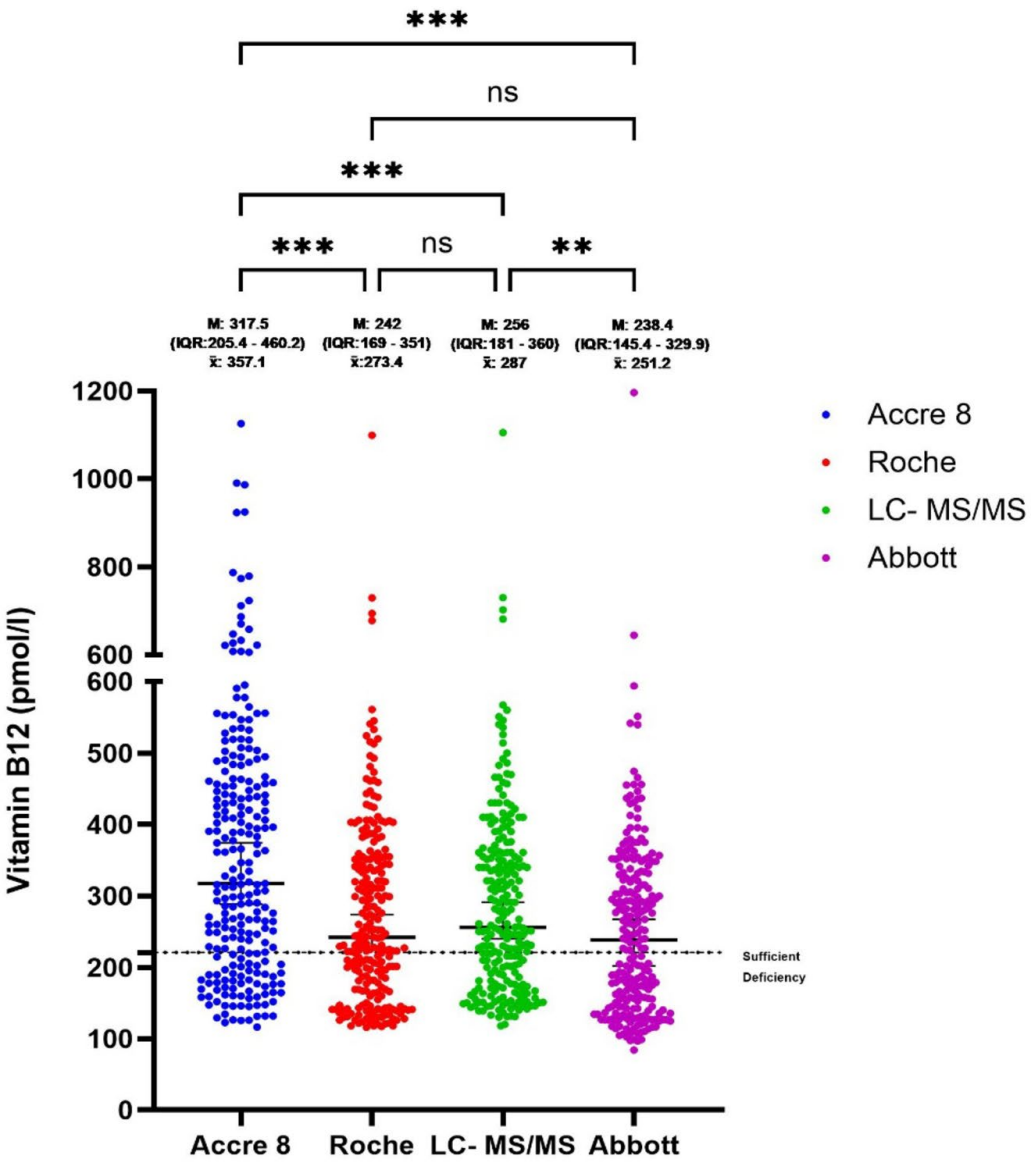
Comparative Vit B12 Level Assessment Across Assays. This scatter plot displays the distribution of Vit B12 (pmol/L) concentrations measured by Accre 8, Roche, LC-MS/MS, and Abbott assays for 297 participants. Each dot represents an individual sample measurement. The black horizontal bars indicate the median (M) values, with the interquartile range (IQR) shown in parentheses, while mean (x̄) values are displayed below the IQR for reference. Participants are categorized as sufficient (> 221 pmol/L) or deficient (≤ 221 pmol/L), with the deficiency threshold represented by a dashed horizontal line for clinical reference. Group differences were analyzed using the Kruskal-Wallis test, followed by pairwise comparisons. Statistical significance is indicated as follows: *p < 0.05, **p < 0.01, ***p < 0.001, ****p < 0.0001, and ns > 0.9999 for non-significant differences.

The comparative analysis of Vit B12 levels across different measurement methodologies demonstrated significant variability, with data reported as median (M) with interquartile range (IQR) and mean (x̄) values due to the non-normal distribution of the data. As shown in Fig. 3, Accre 8 reported a median Vit B12 concentration of 317.5 pmol/L (IQR: 205.4–460.2) and a mean of 357.1 pmol/L, both of which were significantly higher than Roche (M = 242 pmol/L, IQR: 169–351, x̄ = 273.4 pmol/L, *p* < 0.0001). LC-MS/MS, with a median of 256 pmol/L (IQR: 181–360) and a mean of 287 pmol/L, showed no significant difference compared to Roche (ns), indicating consistent performance between these two methods. However, LC-MS/MS values were significantly higher than Abbott (M = 238.4 pmol/L, IQR: 145.4–329, x̄ = 251.2 pmol/L, *p* < 0.01), suggesting Abbott tends to measure slightly lower Vit B12 concentrations. These findings highlight assay-specific differences, with Roche and LC-MS/MS producing comparable results, while Accre 8 exhibited significantly higher Vit B12 concentrations. In contrast, Abbott reported the lowest median and mean values, indicating a potential tendency toward underestimation in Vit B12 measurement.

### Bland-Altman analysis showed good agreement between all assays despite variations

**Fig. 4:**
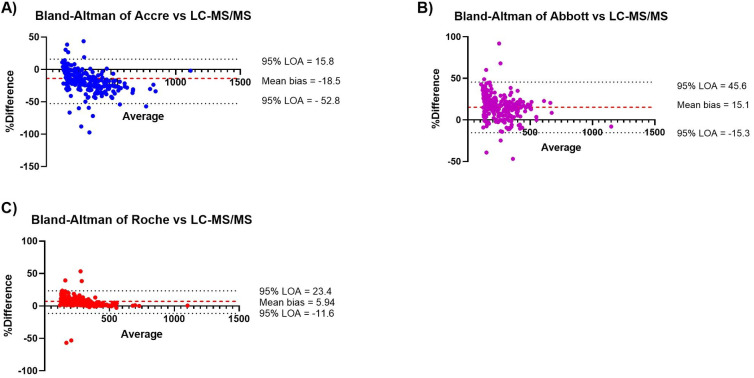
Bland-Altman analysis of Vit B12 levels between different assays. Plots A–C display the mean biases for each comparison, calculated as − 18.5%, 15.1%, and 5.94%, respectively, along with the corresponding 95% limits of agreement (LOA). Plot A shows the comparison between Accre 8 and LC-MS/MS, Plot B compares Abbott and LC-MS/MS, and Plot C presents the comparison between Roche and LC-MS/MS. The mean bias (red dashed line) represents the average difference between the two methods, while the black dotted lines indicate the 95% LOA for each comparison.

Bland-Altman analysis was performed to assess the agreement between Accre 8, Abbott, and Roche assays with LC-MS/MS for Vit B12 measurements (Fig. 4A–C). Figure 4A presents the comparison between Accre 8 and LC-MS/MS, revealing a mean bias of − 18.5% and 95% limits of agreement (LOA) ranging from − 52.8 to 15.8%, indicating a tendency of Accre 8 to underestimate Vit B12 levels relative to LC-MS/MS. Figure 4B illustrates the comparison between Abbott and LC-MS/MS, with a mean bias of 15.1% and 95% LOA between − 15.3% and 45.6%, suggesting that Abbott tends to overestimate Vit B12 concentrations compared to LC-MS/MS. Figure 4C compares Roche and LC-MS/MS, showing the smallest bias among the three assays, with a mean bias of 5.94% and 95% LOA ranging from − 11.6 to 23.4%, indicating the closest alignment with LC-MS/MS. These findings highlight assay-specific differences in measurement trends, with Accre 8 underestimating, Abbott overestimating, and Roche demonstrating the best agreement with LC-MS/MS.

### Passing-Bablok regression reveals proportional Bias in accre 8 at higher concentrations

**Fig. 5:**
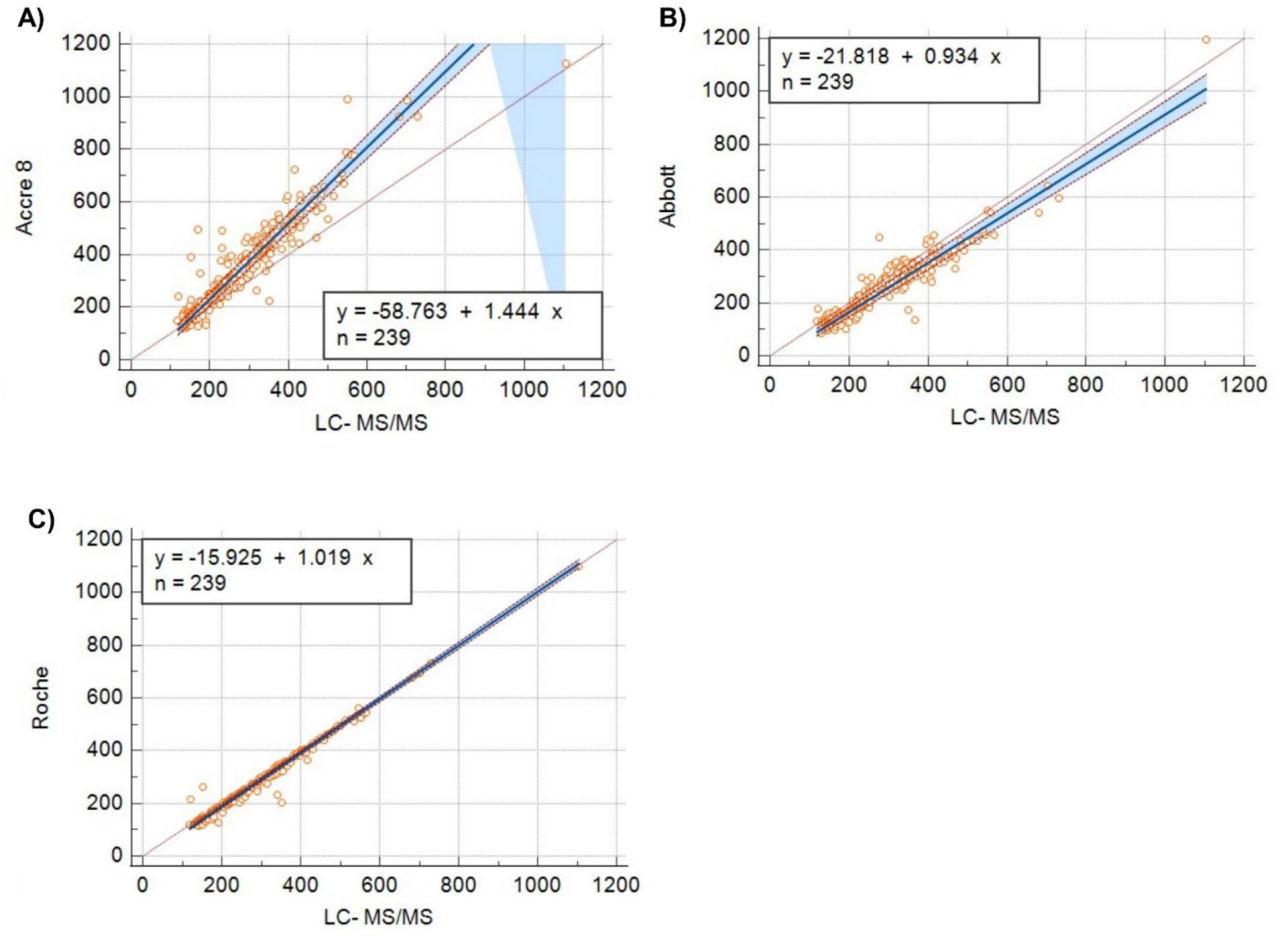
**Passing-Bablock Regression Analysis Comparing Accre 8**,** Abbott**,** and Roche Analysis with LC-MS/MS for Vit B12 Measurements**. Plots (A–C) illustrate the relationship between each assay and LC-MS/MS using Passing-Bablok regression analysis. Plot A presents the regression equation for Accre 8 vs. LC-MS/MS as y = − 58.763 + 1.444x (*n* = 239), indicating a proportional bias where Accre 8 overestimates Vit B12 at higher concentrations. Plot B compares Abbott vs. LC-MS/MS, yielding the equation y = − 21.818 + 0.934x (*n* = 239), showing a slight underestimation of Vit B12 by Abbott. Plot C illustrates Roche vs. LC-MS/MS, with the equation y = − 15.925 + 1.019x (*n* = 239), demonstrating the closest agreement with LC-MS/MS. Orange circles represent individual sample measurements, with LC-MS/MS values on the X-axis plotted against the corresponding Accre 8, Abbott, or Roche values on the Y-axis. The black solid line represents the Passing-Bablok regression fit, indicating the level of agreement between each method and LC-MS/MS. The red dashed line is the identity line (y = x), where perfect agreement would occur. The blue shaded region represents the 95% confidence interval (CI) for the regression line, with a wider spread at higher concentrations, suggesting increased variability and uncertainty in measurement agreement. The thin dashed lines surrounding the regression fit define the confidence limits for the slope.

Passing-Bablok regression analysis was performed to evaluate the agreement between Accre 8, Abbott, and Roche in comparison to LC-MS/MS (Fig. 5A–C). The regression slope for Accre 8 vs. LC-MS/MS was 1.44, indicating a proportional bias, where Accre 8 overestimates Vit B12 concentrations at higher levels (Fig. 5A). In contrast, the slope for Abbott vs. LC-MS/MS was 0.93, suggesting that Abbott slightly underestimates Vit B12 measurements (Fig. 5B). For Roche vs. LC-MS/MS, the regression slope was 1.02, demonstrating the closest agreement with LC-MS/MS and minimal proportional bias (Fig. 5C). These results highlight assay-specific differences in measurement trends, where Accre 8 tends to overestimate at higher concentrations, Abbott shows slight underestimation, and Roche aligns most closely with LC-MS/MS.

### Accre 8 demonstrated excellent diagnostic accuracy compared to LC-MS/MS as well as other assays

**Fig. 6:**
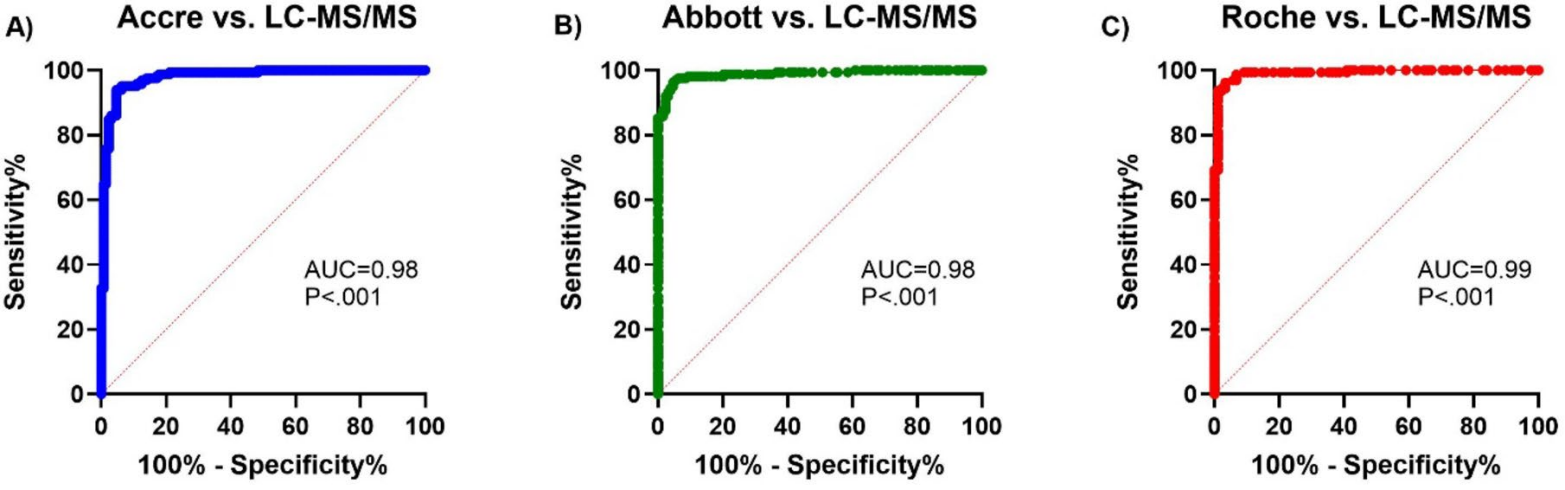
**ROC curve analysis for Accre**,** Roche**,** LC-MS/MS**,** and Abbott.** Graphs [A–C] illustrate the diagnostic performance of Accre, Abbott, and Roche assays in comparison to LC-MS/MS for Vitamin B12 quantification. Panel A presents the ROC curve for Accre 8 vs. LC-MS/MS, yielding an AUC of 0.98 (*P* < 0.001). Panel B shows the ROC curve for Abbott vs. LC-MS/MS, also with an AUC of 0.98 (*P* < 0.001). Panel C displays the ROC curve for Roche vs. LC-MS/MS, which exhibited the highest AUC of 0.99 (*P* < 0.001), indicating the closest agreement with the reference method. AUC values between 0.9 and 1.0 are considered excellent, demonstrating high diagnostic accuracy across all assays.

The ROC curve analysis comparing the diagnostic performance of Accre 8, Abbott, and Roche against LC-MS/MS demonstrated excellent accuracy across all assays, with AUC values ranging from 0.98 to 0.99, as shown in Fig. 6. In Fig. 6A, the comparison between Accre 8 and LC-MS/MS yielded an AUC of 0.98 (*P* < 0.001), indicating a high level of agreement. Similarly, Fig. 6B presents the comparison between Abbott and LC-MS/MS, which also demonstrated an AUC of 0.98 (*P* < 0.001), reflecting strong diagnostic accuracy. Figure 6C displays the comparison between Roche and LC-MS/MS, which exhibited the highest AUC of 0.99 (*P* < 0.001), suggesting the closest alignment with the reference method. These findings confirm that all three assays exhibit excellent diagnostic performance for Vit B12 quantification, with Roche showing the strongest agreement, followed by Accre 8 and Abbott.

### Accre 8 demonstrates strong agreement with LC-MS/MS, second only to Roche

The diagnostic performance of Abbott, Accre 8, and Roche was evaluated in comparison with LC-MS/MS, using key metrics such as sensitivity, specificity, PPV, NPV, and Cohen’s Kappa coefficient (Table 1). These measures provide a comprehensive assessment of each assay’s reliability and agreement with the reference standard. When compared to LC-MS/MS, Abbott demonstrated a sensitivity of 77.7% (CI: 69.1–84.4%) and a specificity of 100% (CI: 97.1–100%), achieving a Cohen’s Kappa of 0.78 (CI: 0.71–0.86), indicating substantial agreement. The PPV was 100% (CI: 95.8–100%), while the NPV was 100% (CI: 76.8–88.6%), reflecting its ability to correctly classify deficient and sufficient Vit B12 levels. Accre 8, in comparison with LC-MS/MS, exhibited a sensitivity of 96.9% (CI: 89.6–99.5%) and a specificity of 86.7% (CI: 80.8–90.9%), achieving a Cohen’s Kappa of 0.76 (CI: 0.67–0.84), suggesting strong agreement. Additionally, the PPV was 73.6% (CI: 63.4–81.7%), while the NPV was 98.7% (CI: 95.3–99.8%), indicating high diagnostic accuracy. When comparing Roche with LC-MS/MS, the assay demonstrated a sensitivity of 88.7% (CI: 80.8–93.6%) and a specificity of 99.3% (CI: 96.1–99.9%), with a PPV of 98.9% (CI: 93.8–99.9%) and an NPV of 92.8% (CI: 87.5–95.9%). The Cohen’s Kappa coefficient of 0.80 (CI: 0.70–0.91) reflects the highest level of agreement with LC-MS/MS among the three assays, reinforcing its reliability as a reference method. These results suggest that while Roche exhibited the highest agreement with LC-MS/MS, Accre 8 demonstrated comparable performance with strong sensitivity and specificity, making it a reliable alternative for Vit B12 quantification. Abbott showed perfect specificity but lower sensitivity, potentially leading to an increased likelihood of false negatives.

**Table 1:** Diagnostic performance of assays in Vit B12 quantification.

Reference	Test	Specificity (%)	Sensitivity (%)	PPV (%)	NPV (%)	Cohen’s Kappa Coefficient
				(CI: 95%)		
**LC-MS/MS**	**Abbott**	100% (97.1–100)	77.7% (69.1–84.4)	100% (95.8–100)	100% (76.8–88.6)	0.78 (0.71–0.86)
**LC-MS/MS**	**Accre 8**	86.7% (80.8–90.9)	96.9% (89.6–99.5)	73.6% (63.4–81.7)	98.7% (95.3–99.8)	0.76 (0.67–0.84)
**LC-MS/MS**	**Roche**	99.3% (96.1–99.9)	88.7% (80.8–93.6)	98.9% (93.8–99.9)	92.8 (87.5–95.9)	0.80 (0.70–0.91)

## Discussion

Many CLIA assays have been developed, but Accre 8 is one of the few designed as a POC system that is currently available on the market. While several CLIA-based assays are available, Accre 8 is one of the few designed as a point-of-care (POC) system, offering rapid testing with minimal calibration. The performance of Accre 8, particularly in comparison with LC-MS/MS and other automated CLIAs (Roche and Abbott), remains a crucial area of validation. The results of this study provide insight into the diagnostic potential of Accre 8 and its comparability to standard laboratory-based assays.

Accre 8 reported the highest median (317.5 pmol/L, IQR: 205.4–460.2) and mean (357.1 pmol/L), significantly higher than Roche and LC-MS/MS, suggesting a potential overestimation at higher Vit B12 concentrations (Fig. 3). Bland-Altman analysis quantified systematic biases across assays and revealed a mean bias of − 18.5% with LOA ranging from − 52.8 to 15.8% for Accre 8 vs. LC-MS/MS, indicating a slight underestimation of Vit B12 levels compared to LC-MS/MS (Fig. 4A). Despite this bias, Accre 8 maintained high diagnostic accuracy, with an AUC of 0.98 in ROC curve analysis (Fig. 6A), demonstrating its strong ability to distinguish between deficient and sufficient Vit B12 levels. Accre 8’s high sensitivity (96.9%) and specificity (86.7%) suggest that its underestimation of Vit B12 levels does not significantly impact clinical assessments, as it effectively identifies true positive and true negative cases (Table 1). Passing-Bablok regression analysis further evaluated systematic and proportional bias for Accre 8 vs. LC-MS/MS, revealing a slope of 1.44, indicating that Accre 8 overestimates Vit B12 concentrations at higher levels (Fig. 5A). This pattern was consistent with Bland-Altman analysis, highlighting the importance of careful interpretation of elevated Vit B12 values when using Accre 8. Accre 8 exhibited a strong correlation with LC-MS/MS (*r* = 0.94, Fig. 2A), which, while slightly lower than Roche’s correlation with LC-MS/MS (*r* = 0.98, Fig. 2C), still indicates a high level of agreement and a reliable relationship between the two methods. Accre 8 overestimation (Fig. 2A) is unlikely to affect clinical assessments, as elevated Vit B12 levels are not toxic^47^. It likely arises from methodological differences, with LC-MS/MS directly quantifying B12, while immunoassays rely on antibody-antigen interactions. Despite this, Accre 8 maintains a robust correlation with LC-MS/MS, reinforcing its diagnostic utility. Furthermore, the strong correlation between Accre 8 and both Roche and Abbott (*r* = 0.94, Fig. 2D and E) further supports its reliability as a CLIA-based assay for Vit B12 quantification. This consistency across multiple platforms highlights its potential as a practical alternative for routine clinical use, ensuring comparable performance to established laboratory-based methods.

Abbott demonstrated a strong correlation with LC-MS/MS (*r* = 0.94) but exhibited notable variability in Vit B12 quantification (Fig. 2B). While Abbott achieved perfect specificity (100%), correctly classifying nearly all true negatives, its lower sensitivity (77.7%) suggests a higher likelihood of false negatives, as slightly reflected in the NPV (100%), indicating a reduced ability to correctly classify non-deficient cases (Table 1). Bland-Altman analysis revealed a mean bias of 15.1% and wide LOA (− 15.3 − 45.6%), further emphasizing Abbott’s measurement variability, particularly at higher concentrations (Fig. 4B). Passing-Bablok regression analysis showed a slope < 1 (Fig. 5B), confirming that Abbott systematically underestimates B12 levels, which could lead to misdiagnosed deficiency cases, especially for borderline results. Additionally, Abbott reported the lowest B12 concentrations (M = 238.4 pmol/L, IQR: 145.4–329, x̄ = 251.2 pmol/L), also indicating a systematic underestimation of B12 levels (Fig. 3). Previous studies have reported that Abbott tends to consistently produce lower B12 values compared to other reference methods such as Roche and Abbott, leading to false-positive deficiency diagnoses. This trend has also been observed in Vit D measurement, particularly in healthy individuals and hemodialysis patients^48^.

Roche demonstrated strong alignment with LC-MS/MS, reporting a median Vit B12 concentration of 242.0 pmol/L (IQR: 169–351) and an average of 273.4 pmol/L (Fig. 3). With a mean bias of just 5.94% and the narrowest limits of agreement (− 11.6–23.4%), it exhibited the highest level of consistency among the three assays, further solidifying its reliability as a reference method (Fig. 4C). However, measurement variability increased at higher Vit B12 concentrations, as seen in the widening LOA in Bland-Altman plots, indicating a decline in assay precision at elevated levels (Fig. 4C). Passing-Bablok regression demonstrated a strong linear relationship with LC-MS/MS, with a slope of 1.01, confirming its accuracy across the measurement range (Fig. 5C). Additionally, findings from Table 1 highlight its high diagnostic agreement, with a sensitivity of 88.7%, specificity of 99.3%, and a Cohen’s Kappa of 0.80, further reinforcing its reliability as a reference method.

The measurement range for each assay was a critical factor in the analysis of the data. Accre 8’s Vit B12 assay demonstrated a distinct advantage in our analyses with its broad measurement range of 48.5–1543.8 pmol/L. Since LC-MS/MS does not operate with defined detection limits, it was able to accurately measure serum samples within the range of 107 to 1801 pmol/L. Roche had a measurement range of 111–1476 pmol/L, which limited its ability to capture data points outside this range. Similarly, Abbott’s range (61–1476 pmol/L), while slightly broader, introduced variability that could misalign with other assays. Such inconsistencies, particularly in Roche and Abbott, underscore the importance of standardization to avoid variability that could affect clinical decisions, especially near diagnostic thresholds.

The Accre 8 assay, a POC CLIA, presents a promising alternative to traditional reference assays for Vit B12 testing. Its compact design, minimal calibration, and ease of use make it suitable for hospitals, outpatient clinics, and resource-limited settings, where quick and reliable results are crucial for patient management. In contrast, while LC-MS/MS is the gold standard, it involves complex sample preparation steps such as liquid-liquid extraction and derivatization, which can introduce variability into results^27^.

This study highlights Accre 8’s clinical utility, demonstrating its potential to streamline diagnostic workflows, reduce treatment delays, and improve patient outcomes. As a POC system, Accre 8 provides rapid results, making it ideal for decentralized healthcare environments where immediate diagnostics are crucial. A review of Accre 8 machines and similar POC CLIA devices supports its diagnostic reliability, adaptability, and feasibility for hospital integration, reinforcing its value as a practical alternative for Vit B12 testing. Moreover, Accre 8 is highly beneficial in clinical settings, particularly in antenatal care, gynecology polyclinics, small laboratory setups, and neonatal screening, where fast, accessible Vit B12 testing is critical for early intervention. Its affordability compared to other assays makes it an attractive option for resource-limited settings, providing cost-effective and reliable diagnostics without the need for high-end automation. However, several practical limitations may affect its real-world application. The need for frequent calibration and quality control checks could introduce workflow challenges, while manual intervention during operations requires user training, unlike fully automated systems^49^. Additionally, unit standardization remains an issue, as Accre 8 reports results in pg/mL, differing from standard laboratory analyzers, increasing the risk of misinterpretation without proper protocols.

Measurement variability in Accre 8 may stem from instrument precision, analytical sensitivity, and assay limitations. As a POC device, its sensitivity differs from high-throughput CLIAs, contributing to greater variability^50^. Pre-analytical factors, such as sample handling, storage, and freeze-thaw cycles, impact results. Improper collection techniques, variations in collection tubes, and clotting or hemolysis can alter biomarker stability^51^. Abbott’s systematic underestimation of B12 levels, as seen in Passing-Bablok regression and Bland-Altman analysis, may be linked to differences in sample handling, calibration, and its microparticle-based CLIA method, in contrast to Roche’s fully automated electrochemiluminescence and Accre 8’s enzymatic CLIA. Analytical factors like person-to-person variability, manual pipetting, instrument calibration, and reagent differences affect precision and can introduce errors. Accre 8, though not fully automated, offers flexibility and accessibility, making it well-suited for point-of-care (POC) testing and rapid diagnostics. Its design allows for on-site testing without the need for complex lab infrastructure, providing convenience and quick turnaround times. Additionally, variations in assay standardization and reference ranges across manufacturers further contribute to measurement discrepancies. Accre 8 follows CLSI EP05-A2 guidelines for interference testing with no significant effects detected, though higher levels or human anti-mouse antibodies (HAMA) could introduce variability, emphasizing the importance of ongoing quality control^32^. Lastly, lipemic (fatty) and hemolyzed samples can interfere with Vit B12 measurements, potentially leading to inaccurate results. Lipemia can cause light scattering or reagent interference in certain assays, while hemolysis releases intracellular Vit B12 and hemoglobin, which may artificially elevate or alter measured concentrations^52,53^. Moreover, samples with high Vit B12 concentrations may introduce a source of variability due to low antibody availability in the assay, potentially saturating binding sites and affecting accurate quantification. This highlights the need for careful assay calibration and quality control measures to ensure reliable performance across a broad concentration range.

This study has several limitations. One major limitation is the absence of a universal cut-off for Vit B12 deficiency, which complicates diagnosis and emphasizes the need for standardization. To address this, we used the WHO cut-off of 221 pmol/L. Additionally, random sampling was not implemented, which may limit the generalizability of the findings. However, a wide range of Vit B12 levels was included using the Roche reference assay to ensure diverse diagnostic profiles. Another limitation of this study is the absence of clinical data to identify potential causes of extreme Vit B12 levels in certain participants, which may affect the interpretation of elevated values and the accuracy of assay performance at high concentrations. Further comparisons with other POC systems, such as the Mini Vidas (Biomerieux)^54^, could provide a deeper understanding of Accre 8’s performance relative to similar platforms. Notably, to our knowledge, Accre 8 is a more cost-effective option compared to the Mini Vidas, making it a potentially more accessible POC testing tool for resource-limited settings.

## Conclusion

The Accre 8 POC analyzer demonstrated excellent diagnostic performance, with high sensitivity, specificity, and strong agreement with LC-MS/MS, making it a reliable option for Vit B12 quantification. Despite a slight underestimation at lower concentrations, Passing-Bablok regression and Bland-Altman analysis confirmed its consistency, with a proportional bias that does not compromise clinical assessments. These findings underscore the importance of selecting accurate and accessible Vit B12 measurement techniques to enhance diagnostic precision and patient care. The Accre 8 POC system presents a promising alternative for rapid and reliable Vit B12 assessment. With further validation to optimize performance across different clinical settings, Accre 8 could be effectively integrated as a standard POC approach for efficient Vit B12 deficiency diagnosis and monitoring.

## Electronic supplementary material

Below is the link to the electronic supplementary material.

Supplementary Material 1

## Data Availability

The original contributions presented in this study are included in the article/supplementary material. Further inquiries can be directed to the corresponding author(s).

## References

[1] Allen, L. H. & Vitamin, B. *Adv. Nutr.* **3**, 10.3945/an.111.001370 (2012/01/01).

[2] JV, P. et al. Transcobalamin receptor antibodies in autoimmune vitamin B12 central deficiency - PubMed. *Sci. Transl. Med.* **16**10.1126/scitranslmed.adl3758 (2024). 10.1126/scitranslmed.adl3758 PMC11520464 38924428

[3] T, W., WC, A. M., L, F. & W. & Vegetarian and vegan diets: benefits and drawbacks - PubMed. *Eur. Heart J.* **44**10.1093/eurheartj/ehad436 (2023). 10.1093/eurheartj/ehad436 PMC10516628 37450568

[4] J, C. R. & V, A. A, D.-L., J, C.-S. Maternal vitamin B12 status during pregnancy and early infant neurodevelopment: the ECLIPSES Study - PubMed. *Nutrients* **15**, 10.3390/nu15061529 (03/22/2023). 10.3390/nu15061529 PMC10051123 36986259

[5] Langan, R. C. G. A. Vitamin B12 Deficiency: Recognition and Management. *Am Fam Physician.*, doi:PMID: 28925645 (2017). 28925645

[6] Shipton, M. J. & Thachil, J. Vitamin B12 deficiency - A 21st century perspective. *Clin. Med. (Lond)*. **15**, 145–150. 10.7861/clinmedicine.15-2-145 (2015). 25824066 10.7861/clinmedicine.15-2-145 PMC4953733

[7] İspir, E. et al. Comparison of four automated serum vitamin B12 assays. *Clin. Chem. Lab. Med. (CCLM)* **53**, 10.1515/cclm-2014-0843 (2015-07-01). 10.1515/cclm-2014-0843 25720078

[8] Szczuko, M., Hawryłkowicz, V., Kikut, J. & Drozd, A. The implications of vitamin content in the plasma in reference to the parameters of carbohydrate metabolism and hormone and lipid profiles in PCOS. *J. Steroid Biochem. Mol. Biol.* **198**, 105570. 10.1016/j.jsbmb.2019.105570 (2020). 31883924 10.1016/j.jsbmb.2019.105570

[9] Knight, B. A. et al. Lower Circulating B12 is associated with higher obesity and insulin resistance during pregnancy in a Non-Diabetic white British population. *PLoS One*. **10**, e0135268. 10.1371/journal.pone.0135268 (2015). 26288227 10.1371/journal.pone.0135268 PMC4545890

[10] Mütze, U. et al. Health outcomes of infants with vitamin B(12) deficiency identified by newborn screening and early treated. *J. Pediatr.* **235**, 42–48. 10.1016/j.jpeds.2021.02.009 (2021). 33581104 10.1016/j.jpeds.2021.02.009

[11] Aparicio-Ugarriza, R., Palacios, G., Alder, M. & González-Gross, M. A review of the cut-off points for the diagnosis of vitamin B12 deficiency in the general population. *Clin. Chem. Lab. Med. (CCLM)*. **53**, 1149–1159. 10.1515/cclm-2014-0784 (2015). 25470607 10.1515/cclm-2014-0784

[12] Green, R. et al. Vitamin B12 deficiency. *Nat. Reviews Disease Primers*. **3**, 17040. 10.1038/nrdp.2017.40 (2017). 28660890 10.1038/nrdp.2017.40

[13] Sturgeon, C. M. & Viljoen, A. Analytical error and interference in immunoassay: minimizing risk. *Ann. Clin. Biochem.* **48**, 418–432. 10.1258/acb.2011.011073 (2011). 21750113 10.1258/acb.2011.011073

[14] Ward, G., Simpson, A., Boscato, L. & Hickman, P. E. The investigation of interferences in immunoassay. *Clin. Biochem.* **50**, 1306–1311. 10.1016/j.clinbiochem.2017.08.015 (2017). 28847718 10.1016/j.clinbiochem.2017.08.015

[15] Wauthier, L., Plebani, M. & Favresse, J. Interferences in immunoassays: review and practical algorithm. *Clin. Chem. Lab. Med. (CCLM)*. **60**, 808–820. 10.1515/cclm-2021-1288 (2022). 35304841 10.1515/cclm-2021-1288

[16] Ho, M. et al. Vitamin B12 in obese adolescents with clinical features of insulin resistance. *Nutrients* **6**, 5611–5618 (2014). 25486369 10.3390/nu6125611 PMC4276987

[17] Snellman, G., Gedeborg, M. H., Byberg, R., Berglund, L. & Wernroth, L. L, et al. Determining vitamin D status: A comparison between commercially available assays. *PLoS ONE* **5**(7): e11555 ., 10.1371/journal.pone.0011555 ((2010)). 10.1371/journal.pone.0011555 PMC2903481 20644628

[18] Lee, J. H., Choi, J. H., Kweon, O. J. & Park, A. J. Discrepancy between vitamin D total immunoassays due to various Cross-reactivities. *J. Bone Metab.* **22**, 107–112. 10.11005/jbm.2015.22.3.107 (2015). 26389085 10.11005/jbm.2015.22.3.107 PMC4572031

[19] Xiao, Q. & Xu, C. Research progress on chemiluminescence immunoassay combined with novel technologies. *TRAC Trends Anal. Chem.* **124**, 115780. 10.1016/j.trac.2019.115780 (2020).

[20] Johan, S., Ellen, A. & Johan, S. in *Advances in Immunoassay Technology* (eds H. L. Chiu Norman & K. Christopoulos Theodore) Ch. 3IntechOpen, (2012).

[21] S., D. Interferences in quantitative immunochemical methods. *Biochem. Med. (Zagreb)* **19**:50–62 (2009).

[22] J., P. J. Principles and applications of liquid chromatography-mass spectrometry in clinical biochemistry. *Clin. Biochemist Reviews*. **30**, 19–34 (2009). PMC2643089 19224008

[23] C, S. & L, S. After another decade: LC-MS/MS became routine in clinical diagnostics - PubMed. *Clinical biochemistry* 82. 10.1016/j.clinbiochem.2020.03.004 (2020 Aug). 10.1016/j.clinbiochem.2020.03.004 32188572

[24] Junger, S. et al. Automated LC-MS/MS: ready for the clinical routine laboratory?? *J. Mass. Spectrom. Adv. Clin. Lab.* **30**, 10.1016/j.jmsacl.2023.07.001 (2023/11/01). 10.1016/j.jmsacl.2023.07.001 PMC10423925 37583571

[25] Mikulska-Sauermann, A. A., Karaźniewicz-Łada, M., Filipowicz, D., Ruchała, M. & Główka, F. Measurement of serum vitamins B2 and B6 in patients with Hashimoto’s thyroiditis by LC–MS/MS method. *Chromatographia* **87**, 433–443. 10.1007/s10337-024-04319-x (2024).

[26] Chew, Y. L. et al. LC-MS/MS method for sensitive detection and quantitation of 8 Water-Soluble vitamins in infant milk powder.

[27] Nitin Shukla, D. S., Patil, S. & Rasam, P. Quantitative analysis of vitamin B complex in Dietary supplement powder by LC-MS/MS. (2023).

[28] Huang, B., Zhang, J., Wang, M. & Cai, Z. Determination of Vitamin B12 in Milk and Dairy Products by Isotope-Dilution Liquid Chromatography Tandem Mass Spectrometry. *Journal of Food Quality* (2022). 10.1155/2022/7649228 (2022/01/01).

[29] Legoupil, T., Jaffeul, A. & Huteau, A. Shimadzu France,. A novel fast and simple quantification method for vitamins, complements, and contaminants in milk infant formulas by LC-MS/MS., (2017).

[30] Yetim, A. et al. Measurement of serum vitamin B12-related metabolites in newborns: implications for new cutoff values to detect B12 deficiency. *J. Maternal-Fetal Neonatal Med.* **34**, 10.1080/14767058.2019.1633301 (2021-4-18). 10.1080/14767058.2019.1633301 31204544

[31] Basalamah, M. A. et al. Vitamin B12 status among asymptomatic young adult females and its association with some anthropometric and biochemical parameters: A cross-sectional study from Makkah (cobalamin deficiency in young adult females). *Medicine* **102**, e35838. 10.1097/md.0000000000035838 (2023). 37933046 10.1097/MD.0000000000035838 PMC10627631

[32] Shenzhen Tisenc Medical Devices Co. L. (ed Ltd. Shenzhen Tisenc Medical Devices Co., distributed by Shanghai International Holding Corp. GmbH (Europe)).

[33] GmbH, R. D. (ed) (ed Mannheim Roche Diagnostics GmbH, Germany.).

[34] Laboratories, A. *ARCHITECT B12 Assay Package Insert* (Longford, 2015).

[35] KH, Z. & SG, S. K, T. Correlation and simple linear regression - PubMed. *Radiology* **227**, 10.1148/radiol.2273011499 (2003 Jun). 10.1148/radiol.2273011499 12773666

[36] Yassine, H. M. et al. Performance evaluation of five ELISA kits for detecting anti-SARS-COV-2 IgG antibodies. *Int. J. Infect. Dis.* **102**, 181–187. 10.1016/j.ijid.2020.10.042 (2021). 33127504 10.1016/j.ijid.2020.10.042 PMC7590641

[37] Younes, S. et al. Follow up and comparative assessment of IgG, IgA, and neutralizing antibody responses to SARS-CoV-2 between mRNA-vaccinated Naïve and unvaccinated naturally infected individuals over 10 months. *J. Infect. Public Health*. **16**, 1729–1735. 10.1016/j.jiph.2023.08.009 (2023). 37734128 10.1016/j.jiph.2023.08.009

[38] Nasrallah, G. K. et al. Prevalence of syphilis infection among migrant workers in Qatar: a nationwide cross-sectional survey. *BMJ Open.* **14**, e083810. 10.1136/bmjopen-2023-083810 (2024). 39609019 10.1136/bmjopen-2023-083810 PMC11603719

[39] Bland, J. M., Altman, D. G. & Bland, J. M. D. G. A. Measuring agreement in method comparison studies. *Stat. Methods Med. Res.* **8**, 10.1177/096228029900800204 (1999-04-01). 10.1177/096228029900800204 10501650

[40] Younes, N., Al Ghwairi, M. M., Majdalawieh, A. F., Al-Dweik, N. & Nasrallah, G. K. Performance evaluation of new fluorescent-based lateral flow immunoassay for quantification of HbA1c in diabetic patients. *medRxiv*, 2022.2010.2027.22281596, (2022). 10.1101/2022.10.27.22281596 10.31083/j.fbl2803060 37005766

[41] Bilić-Zulle, L. Comparison of methods: passing and Bablok regression. *Biochemia Med.* **21**, 10.11613/BM.2011.010 (2011/02/15). 10.11613/bm.2011.010 22141206

[42] Schoonjans, F., Zalata, A., Depuydt, C. E. & Comhaire, F. H. MedCalc: a new computer program for medical statistics. *Comput. Methods Programs Biomed.* **48**10.1016/0169-2607(95)01703-8 (1995/12/01). 10.1016/0169-2607(95)01703-8 8925653

[43] RB, P. Method comparison: evaluation of least squares, Deming and Passing/Bablok regression procedures using computer simulation - PubMed. *Ann. Clin. Biochem.* **34** (Pt 3), 10.1177/000456329703400317 (1997 May). 10.1177/000456329703400317 9158833

[44] M, L. & T, Y. Methodological issues on evaluating agreement between two detection methods by Cohen’s kappa analysis - PubMed. *Parasites Vectors* **15**, 10.1186/s13071-022-05402-8 (07/29/2022). 10.1186/s13071-022-05402-8 PMC9338630 35906628

[45] Nasrallah, K. Evaluation of the mindray CL900i CLIA HIV Ag/Ab combo assay for sensitive and specific HIV screening compared to established methods. *Sci. Rep.* **14**, 28177. 10.1038/s41598-024-78271-z (2024). 39548153 10.1038/s41598-024-78271-z PMC11568275

[46] Yassine, H. M. et al. Performance evaluation of five ELISA kits for detecting anti-SARS-COV-2 IgG antibodies. *Int. J. Infect. Dis.* **102**, 181–187. 10.1016/j.ijid.2020.10.042 (2021). 33127504 10.1016/j.ijid.2020.10.042 PMC7590641

[47] Supplements, N. O. o. D. Vitamin B12 Fact Sheet for Health Professionals. *NIH* (2024).

[48] B, D., AC, H. & MR, L. Accuracy of three automated 25-hydroxyvitamin D assays in Hemodialysis patients - PubMed. *Clin. Chim. Acta* **415**, 10.1016/j.cca.2012.10.056 (01/16/2013). 10.1016/j.cca.2012.10.056 23159781

[49] Chokkalla, A. K., Recio, B. D. & Devaraj, S. Best Practices for Effective Management of Point of Care Testing. *EJIFCC*, 245–249. (2023). PMC10588082 37868087

[50] Indrasari, N. D., Wonohutomo, J. P. & Sukartini, N. Comparison of point-of‐care and central laboratory analyzers for blood gas and lactate measurements. *J. Clin. Lab. Anal.* **33**, 10.1002/jcla.22885 (2019/06/01). 10.1002/jcla.22885 PMC6595289 30924550

[51] Keukeleire, S. D. et al. Stability of vitamin B12 — A preanalytical view. *Clin. Chim. Acta* **448**, 10.1016/j.cca.2015.06.015 (2015/08/25). 10.1016/j.cca.2015.06.015 26116894

[52] A, K. et al. Evaluation of hemolysis, lipemia, and icterus interference with common clinical immunoassays - PubMed. *Clin. Chem. Lab. Med.* **61**, 10.1515/cclm-2022-0924 (01/13/2023). 10.1515/cclm-2022-0924 36635939

[53] Krasowski, M. D. Educational Case: Hemolysis and Lipemia Interference With Laboratory Testing. *Academic Pathology* 6. 10.1177/2374289519888754 (2019 Nov 22). 10.1177/2374289519888754 PMC6876161 31803827

[54] S.A., b. *bioMérieux S.A. VIDAS*^®^*Vitamin B12 Total: Automated in-House Testing, Adapted to Your Workflow*. (bioMérieux S.A., (2024).

