# Supplementary material for: Accre 8 emerging point of care CLIA system for vitamin B12 assessment compared with three established assays
Source: Sci Rep. 2025 Apr 17;15:13328. doi: 10.1038/s41598-025-97503-4 (PMC12006362; doi:10.1038/s41598-025-97503-4)
Supplement: Supplementary file 1 — Supplementary Material 1 [file 41598_2025_97503_MOESM1_ESM.pdf]

Supplementary Material Images

Accre as an Emerging Point-of-Care CLIA System for Vitamin B12 Assessment: Comparative Analysis with Three Reference Assays

Farah M. Trad[1†], Tasneem AlHamad[2†], Nadin Younes[1,2], Shaden Abunasser[1,2], Salma Younes[1,2], Parveen B. Nizamuddin[1], Dayana El Chaar[3], Israa M. Salameh[1], Nader I. Al-dewik[4], Wanida Laiwattanapaisal[5], Pattramon Aungbamnet[6], Pollanat Loungjinda[6], Palanee Ammaranond[7], Meng Li[8], Laith J. Abu-Raddad[9], Gheyath K. Nasrallah[1,2*]

[1] Biomedical Research Center, QU Health, Qatar University, Doha P.O. Box 2713, Qatar.
[2] Biomedical Sciences Department, College of Health Sciences, QU Health, Qatar University, Doha P.O. Box 2713, Qatar
[3] University of Toronto, Faculty of Medicine, Department of Nutritional Sciences, 27 King's College Cir, Toronto, Ontario M5S 1A1
[4] Department of Research, Women's Wellness and Research Center, Hamad Medical Corporation, P.O.BOX. 3050, Doha, Qatar
[5] Department of Clinical Chemistry, Faculty of Allied Health Sciences, Chulalongkorn University, Patumwan, Bangkok, 10330, Thailand
[6] Medical Technology Unit, Health Sciences Service Center, Faculty of Allied Health Sciences, Chulalongkorn University, Bangkok, Thailand
[7] Department of transfusion medicine and clinical microbiology, Faculty of Allied Health Sciences, Chulalongkorn University, Bangkok 10330, Thailand
[8] Guangzhou Wondfo Biotech Co., Ltd., No. 8 Lizhishan Road, Science City, Huangpu District, Guangzhou 510663, China
[9] Weill Cornell Medical College – Qatar, Cornell University, Qatar Foundation - Education City, Doha, Qatar

†Authors contributed equally

***Correspondence:** Gheyath K. Nasrallah

Department of Biomedical Science, College of Health Sciences, Qatar University, Doha 2713, Qatar. Tel: +974 4403 4817, Email: gheyath.nasrallah@qu.edu.qa

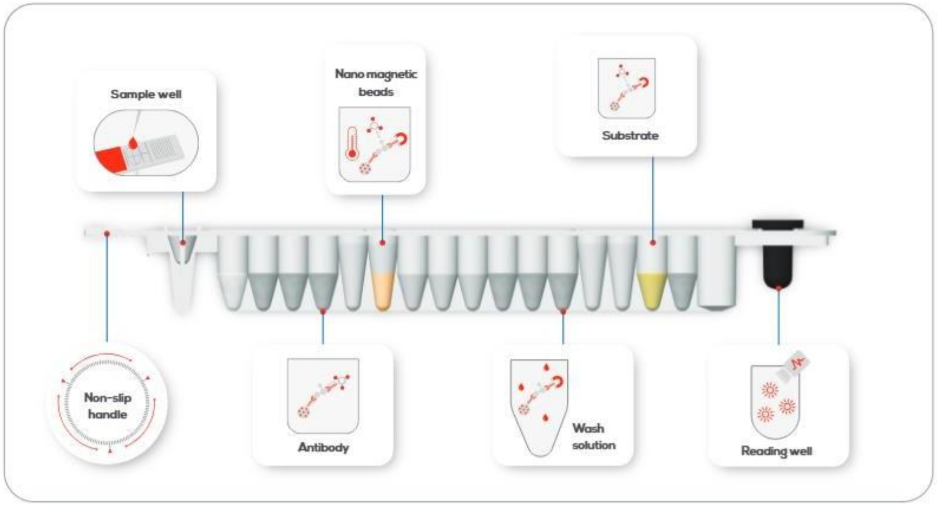

Figure S1 A. Workflow and Components of the Assay System. This figure depicts the key steps and components of the assay system: [1] **Sample Well:** The serum or plasma sample is added here to initiate the assay.[2] **Nano Magnetic Beads:** Antibody-coated beads capture the target analyte from the sample. [3] **Antibody**: Binds specifically to the analyte, forming immune complexes. [4] **Wash Solution**: Removes unbound substances, ensuring assay specificity. [5] **Substrate**: Reacts with the immune complexes to generate a detectable signal. [6] **Reading Well**: The signal is measured, correlating with the analyte concentration.

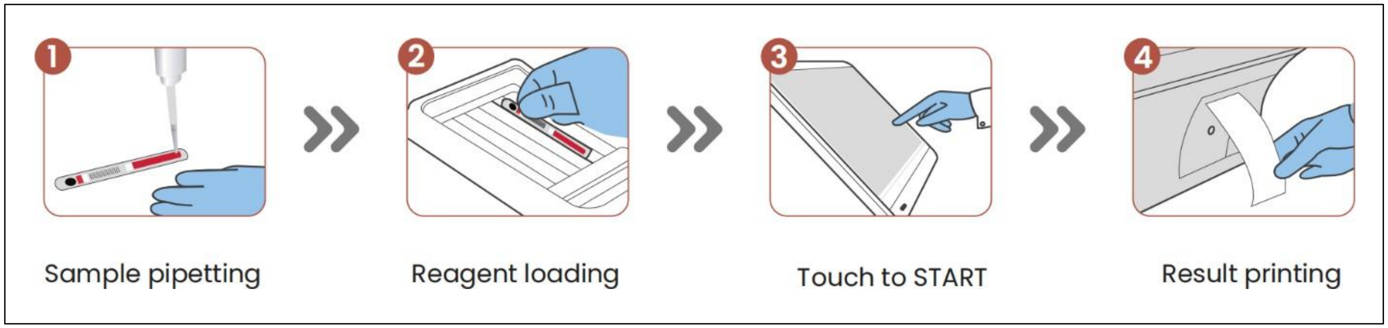

Figure S1 B. Testing workflow of the Accre CLIA POC system. (1) The sample was pipetted onto the reagent strip, (2) The strip was inserted into the device, (3) The 'Start' button was pressed to initiate the assay, and (4) The results were printed.

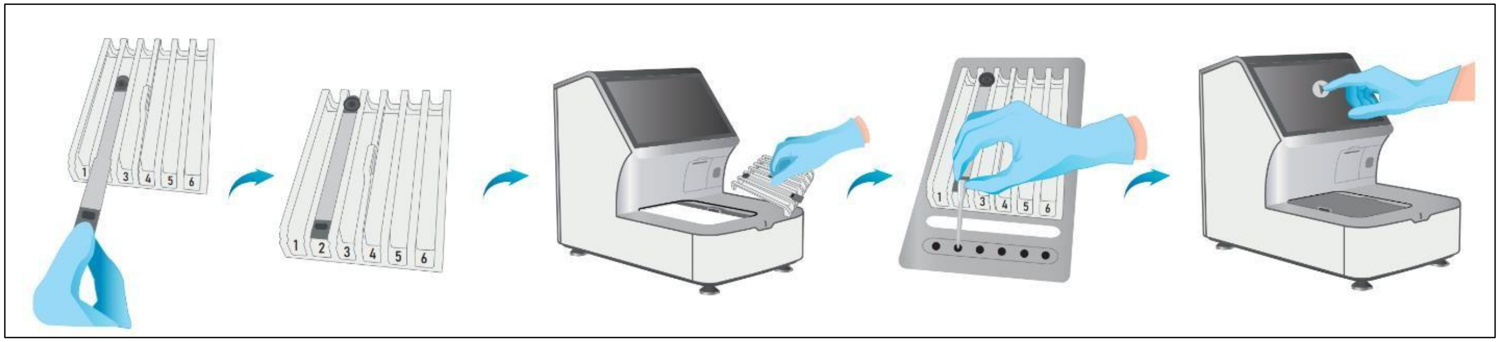

Figure S1 C. Operational workflow of the Accre CLIA POC system. Reagent strip is taken from the storage pack and inserted to the reagent cartridge. Reagent rack is placed into the chamber. Sample is pipetted into the cartridge. Assay tips are placed into the chamber. Sample ID is entered into the software and test is started.

Table S1 A. Intre-Day Imprecision Analysis of Vitamin B12 Test Measurements at Low and High Concentration Levels

Imprecision intre-day

		Test 1	Test 2	Test 3	Test 4	Test 5	Mean
**Day 1**	Low	407.1	317.09	304.56	312.29	357.98	339.804
**Day 2**	Low	439.73	331.89	314.48	297.64	341.01	344.95
**Day 3**	Low	413.62	376.68	293.89	358.93	277.92	344.208
**Day 4**	Low	377.63	373.13	272.29	363.06	294.85	336.192
**Day 5**	Low	382.31	336.91	323.84	328.58	276	329.528
						**Overall mean**	338.9364
						**SD**	6.339472
						**CV**	1.870402

		Test 1	Test 2	Test 3	Test 4	Test 5	Mean
**Day 1**	High	1507	1580.4	1420.2	1493.2	1390.6	1478.28
**Day 2**	High	1477.5	1548.6	1402.2	1410.1	1409.3	1449.54
**Day 3**	High	1481.2	1545.5	1416.8	1467	1418.1	1465.72
**Day 4**	High	1771.3	1569.9	1384.8	1462.6	1413.4	1520.4
**Day 5**	High	1743.9	1585.8	1485	1501.2	1422	1547.58
						**Overall mean**	1492.304
						**SD**	40.54924
						**CV**	2.717224

SD: Standard Deviation; CV: Coefficient of Variation.

Table S1 B. Intra-Day Imprecision of Vitamin B12 Measurements at Low and High Concentration Levels

Imprecision intra-day

	**Low**	**High**
	291.79	1390.6
	303.1	1409.3
	318	1418.1
	298.56	1413.4
	290.87	1422
	357.98	1503.9
	341.01	1516.6
	277.92	1451.8
	294.85	1409.7
	276	1435.6
AV	305.01	1437.1
SD	26.60	41.95
CV	8.72%	2.92%

AV: Average; SD: Standard Deviation; CV: Coefficient of Variation.

Table S2. Comprehensive data of Vitamin B12 levels measured by various assays for all study participants

Comprehensive Data of Vitamin B12 Levels Measured by Various Assays for All Study Participants								
**Lab ID**	**Accre 8**	**Roche**	**LC-MS/MS**	**Architect ci4100**	**Group**	**Nationality**	**Gender**	**Age**
**1**	423.20	384	1110	363.83	Vitamin B-12 -Control (Normal Vitamin B12)	OTHER	MALE	53
**2**	425.27	223	231	295.94	Vitamin B-12 -Control (Normal Vitamin B12)	QATARI	FEMALE	33
**3**	460.24	387	390	288.56	Vitamin B-12 -Control (Normal Vitamin B12)	QATARI	FEMALE	41
**4**	440.48	403	410	277.49	Vitamin B-12 -Control (Normal Vitamin B12)	ARAB	MALE	21
**5**	386.59	267	290	276.01	Vitamin B-12 -Control (Normal Vitamin B12)	ARAB	FEMALE	47
**6**	319.47	274	277	446.49	Vitamin B-12 -Control (Normal Vitamin B12)	ARAB	MALE	30
**7**	440.96	424	427	377.12	Vitamin B-12 -Control (Normal Vitamin B12)	QATARI	MALE	39
**8**	264.57	316	320	354.24	Vitamin B-12 -Control (Normal Vitamin B12)	QATARI	FEMALE	56
**9**	377.51	223	227	226.57	Vitamin B-12 -Control (Normal Vitamin B12)	ARAB	FEMALE	65
**10**	365.13	343	351	304.79	Vitamin B-12 -Control (Normal Vitamin B12)	ARAB	FEMALE	38
**11**	N/A	257	260	291.51	Vitamin B-12 -Control (Normal Vitamin B12)	QATARI	FEMALE	32
**12**	N/A	323	325	329.89	Vitamin B-12 -Control (Normal Vitamin B12)	QATARI	MALE	31
**13**	456.68	314	321	310.70	Vitamin B-12 -Control (Normal Vitamin B12)	OTHER	MALE	22
**14**	N/A	239	246	1196.30	Vitamin B-12 -Control (Normal Vitamin B12)	QATARI	MALE	49
**15**	464.05	462	470	319.55	Vitamin B-12 -Control (Normal Vitamin B12)	QATARI	MALE	37
**16**	483.96	356	360	324.72	Vitamin B-12 -Control (Normal Vitamin B12)	QATARI	MALE	31
**17**	1125.97	1099	1105	455.35	Vitamin B-12 -Control (Normal Vitamin B12)	QATARI	MALE	67
**18**	418.80	318	321	379.33	Vitamin B-12 -Control (Normal Vitamin B12)	QATARI	FEMALE	67
**19**	463.07	294	296	302.58	Vitamin B-12 -Control (Normal Vitamin B12)	QATARI	MALE	55
**20**	723.14	392	415	354.24	Vitamin B-12 -Control (Normal Vitamin B12)	ARAB	MALE	37
**21**	556.02	403	410	279.70	Vitamin B-12 -Control (Normal Vitamin B12)	ARAB	MALE	32
**22**	431.03	355	361	262.73	Vitamin B-12 -Control (Normal Vitamin B12)	ARAB	MALE	44
**23**	519.60	355	361	192.62	Vitamin B-12 -Control (Normal Vitamin B12)	QATARI	FEMALE	33
**24**	277.62	271	279	133.58	Vitamin B-12 -Control (Normal Vitamin B12)	QATARI	FEMALE	43
**25**	361.36	276	281	226.57	Vitamin B-12 -Control (Normal Vitamin B12)	QATARI	MALE	58
**26**	265.45	227	245	134.32	Vitamin B-12 -Control (Normal Vitamin B12)	QATARI	MALE	53
**27**	129.47	181	191	156.46	Vitamin B-12 -Case (Deficiency Vitamin B12 Borderline)	QATARI	MALE	42

**28**	305.79	201	222	172.69	Vitamin B-12 -Case (Deficiency Vitamin B12 Borderline)	QATARI	FEMALE	43
**29**	181.95	177	181	142.43	Vitamin B-12 -Case (Deficiency Vitamin B12 Borderline)	QATARI	FEMALE	66
**30**	195.30	188	193	167.53	Vitamin B-12 -Case (Deficiency Vitamin B12 Borderline)	QATARI	FEMALE	59
**31**	214.47	197	210	177.86	Vitamin B-12 -Case (Deficiency Vitamin B12 Borderline)	QATARI	FEMALE	32
**32**	219.00	182	186	189.67	Vitamin B-12 -Case (Deficiency Vitamin B12 Borderline)	QATARI	FEMALE	21
**33**	248.72	187	196	116.60	Vitamin B-12 -Case (Deficiency Vitamin B12 Borderline)	QATARI	MALE	29
**34**	247.66	201	203	152.77	Vitamin B-12 -Case (Deficiency Vitamin B12 Borderline)	QATARI	FEMALE	44
**35**	259.17	210	226	178.60	Vitamin B-12 -Case (Deficiency Vitamin B12 Borderline)	QATARI	MALE	39
**36**	176.06	143	167	184.50	Vitamin B-12 -Case (Deficiency Vitamin B12 Deficiency)	QATARI	FEMALE	20
**37**	226.32	188	205	128.41	Vitamin B-12 -Case (Deficiency Vitamin B12 Borderline)	QATARI	MALE	29
**38**	275.92	210	230	177.86	Vitamin B-12 -Case (Deficiency Vitamin B12 Borderline)	QATARI	MALE	45
**39**	227.75	196	206	140.22	Vitamin B-12 -Case (Deficiency Vitamin B12 Borderline)	QATARI	FEMALE	30
**40**	177.62	147	166	188.93	Vitamin B-12 -Case (Deficiency Vitamin B12 Deficiency)	QATARI	MALE	44
**41**	290.30	209	226	132.84	Vitamin B-12 -Case (Deficiency Vitamin B12 Borderline)	QATARI	MALE	49
**42**	176.94	139	156	157.93	Vitamin B-12 -Case (Deficiency Vitamin B12 Deficiency)	QATARI	FEMALE	32
**43**	293.10	206	220	184.50	Vitamin B-12 -Case (Deficiency Vitamin B12 Borderline)	QATARI	FEMALE	34
**44**	326.82	166	176	96.68	Vitamin B-12 -Case (Deficiency Vitamin B12 Borderline)	QATARI	MALE	53

**45**	237.49	182	201	182.29	Vitamin B-12 -Case (Deficiency Vitamin B12 Borderline)	QATARI	FEMALE	35
**46**	284.75	216	231	110.70	Vitamin B-12 -Case (Deficiency Vitamin B12 Borderline)	QATARI	MALE	36
**47**	164.62	122	146	115.87	Vitamin B-12 -Case (Deficiency Vitamin B12 Deficiency)	QATARI	MALE	61
**48**	285.93	194	210	98.89	Vitamin B-12 -Case (Deficiency Vitamin B12 Borderline)	QATARI	FEMALE	44
**49**	190.52	141	167	126.94	Vitamin B-12 -Case (Deficiency Vitamin B12 Deficiency)	QATARI	MALE	57
**50**	182.56	164	181	157.93	Vitamin B-12 -Case (Deficiency Vitamin B12 Borderline)	ARAB	FEMALE	35
**51**	151.98	144	163	189.67	Vitamin B-12 -Case (Deficiency Vitamin B12 Deficiency)	QATARI	MALE	31
**52**	187.53	172	200	126.20	Vitamin B-12 -Case (Deficiency Vitamin B12 Borderline)	QATARI	FEMALE	53
**53**	260.14	197	220	124.72	Vitamin B-12 -Case (Deficiency Vitamin B12 Borderline)	QATARI	MALE	43
**54**	242.03	220	246	121.03	Vitamin B-12 -Case (Deficiency Vitamin B12 Borderline)	QATARI	FEMALE	37
**55**	200.65	160	173	162.36	Vitamin B-12 -Case (Deficiency Vitamin B12 Borderline)	QATARI	MALE	51
**56**	495.04	160	171	144.65	Vitamin B-12 -Case (Deficiency Vitamin B12 Borderline)	QATARI	FEMALE	38
**57**	158.10	155	173	226.57	Vitamin B-12 -Case (Deficiency Vitamin B12 Borderline)	QATARI	MALE	49
**58**	254.65	165	202	124.72	Vitamin B-12 -Case (Deficiency Vitamin B12 Borderline)	QATARI	FEMALE	41
**59**	253.53	200	220	202.95	Vitamin B-12 -Case (Deficiency Vitamin B12 Borderline)	QATARI	MALE	45
**60**	307.48	214	251	168.26	Vitamin B-12 -Case (Deficiency Vitamin B12 Borderline)	QATARI	FEMALE	46
**61**	169.52	139	153	172.69	Vitamin B-12 -Case (Deficiency Vitamin B12 Deficiency)	ARAB	MALE	44

**62**	488.47	205	230	126.94	Vitamin B-12 -Case (Deficiency Vitamin B12 Borderline)	QATARI	FEMALE	22
**63**	249.61	178	200	197.05	Vitamin B-12 -Case (Deficiency Vitamin B12 Borderline)	QATARI	FEMALE	53
**64**	225.53	203	351	174.17	Vitamin B-12 -Case (Deficiency Vitamin B12 Borderline)	QATARI	MALE	36
**65**	131.81	150	152	163.10	Vitamin B-12 -Case (Deficiency Vitamin B12 Borderline)	QATARI	FEMALE	32
**66**	248.97	199	216	123.25	Vitamin B-12 -Case (Deficiency Vitamin B12 Borderline)	QATARI	MALE	48
**67**	259.89	208	216	183.02	Vitamin B-12 -Case (Deficiency Vitamin B12 Borderline)	QATARI	MALE	36
**68**	217.89	169	176	128.41	Vitamin B-12 -Case (Deficiency Vitamin B12 Borderline)	QATARI	FEMALE	73
**69**	164.69	156	386	178.60	Vitamin B-12 -Case (Deficiency Vitamin B12 Borderline)	QATARI	FEMALE	51
**70**	219.54	220	222	190.40	Vitamin B-12 -Case (Deficiency Vitamin B12 Borderline)	QATARI	FEMALE	50
**71**	170.09	169	171	187.45	Vitamin B-12 -Case (Deficiency Vitamin B12 Borderline)	QATARI	FEMALE	30
**72**	239.67	215	120	205.16	Vitamin B-12 -Case (Deficiency Vitamin B12 Borderline)	QATARI	FEMALE	36
**73**	248.55	208	520	204.43	Vitamin B-12 -Case (Deficiency Vitamin B12 Borderline)	QATARI	FEMALE	29
**74**	264.23	204	246	143.17	Vitamin B-12 -Case (Deficiency Vitamin B12 Borderline)	QATARI	FEMALE	55
**75**	N/A	127	205	174.91	Vitamin B-12 -Case (Deficiency Vitamin B12 Deficiency)	ARAB	MALE	55
**76**	250.93	198	210	150.55	Vitamin B-12 -Case (Deficiency Vitamin B12 Borderline)	QATARI	FEMALE	21
**77**	288.31	201	219	136.53	Vitamin B-12 -Case (Deficiency Vitamin B12 Borderline)	ARAB	MALE	72
**78**	202.37	167	173	107.75	Vitamin B-12 -Case (Deficiency Vitamin B12 Borderline)	QATARI	FEMALE	30

**79**	246.20	194	216	117.34	Vitamin B-12 -Case (Deficiency Vitamin B12 Borderline)	QATARI	MALE	50
**80**	205.39	185	189	290.77	Vitamin B-12 -Case (Deficiency Vitamin B12 Borderline)	QATARI	FEMALE	36
**81**	225.82	211	216	169.00	Vitamin B-12 -Case (Deficiency Vitamin B12 Borderline)	QATARI	FEMALE	29
**82**	156.51	140	145	155.72	Vitamin B-12 -Case (Deficiency Vitamin B12 Deficiency)	QATARI	FEMALE	43
**83**	125.84	167	171	109.96	Vitamin B-12 -Case (Deficiency Vitamin B12 Borderline)	QATARI	FEMALE	26
**84**	402.35	320	355	102.58	Vitamin B-12 -Control (Normal Vitamin B12)	QATARI	FEMALE	37
**85**	211.86	208	210	126.20	Vitamin B-12 -Case (Deficiency Vitamin B12 Borderline)	QATARI	MALE	25
**86**	221.33	169	177	158.67	Vitamin B-12 -Case (Deficiency Vitamin B12 Borderline)	QATARI	FEMALE	37
**87**	181.74	<111	120	112.91	Vitamin B-12 -Case (Deficiency Vitamin B12 Outliers)	QATARI	FEMALE	49
**88**	142.10	<111	126	145.39	Vitamin B-12 -Case (Deficiency Vitamin B12 Outliers)	ARAB	MALE	27
**89**	161.46	140	155	129.15	Vitamin B-12 -Case (Deficiency Vitamin B12 Deficiency)	QATARI	FEMALE	28
**90**	162.52	145	151	76.01	Vitamin B-12 -Case (Deficiency Vitamin B12 Deficiency)	QATARI	FEMALE	19
**91**	102.59	<111	113	354.24	Vitamin B-12 -Case (Deficiency Vitamin B12 Outliers)	QATARI	FEMALE	58
**92**	137.41	<111	130	135.79	Vitamin B-12 -Case (Deficiency Vitamin B12 Outliers)	QATARI	FEMALE	39
**93**	388.87	260	151	122.51	Vitamin B-12 -Control (Normal Vitamin B12)	QATARI	MALE	23
**94**	146.77	117	118	127.67	Vitamin B-12 -Case (Deficiency Vitamin B12 Deficiency)	QATARI	FEMALE	44
**95**	1380.06	>1476	1561	166.05	Vitamin B-12 -Case (Deficiency Vitamin B12 Outliers)	QATARI	FEMALE	25
**96**	101.76	<111	123	97.42	Vitamin B-12 -Case (Deficiency Vitamin B12 Outliers)	QATARI	MALE	54
**97**	555.65	365	416	84.13	Vitamin B-12 -Control (Normal Vitamin B12)	QATARI	FEMALE	32
**98**	202.99	138	165	106.27	Vitamin B-12 -Case (Deficiency Vitamin B12 Deficiency)	QATARI	FEMALE	43
**99**	173.08	128	152	126.20	Vitamin B-12 -Case (Deficiency Vitamin B12 Deficiency)	QATARI	FEMALE	29

**100**	147.56	143	160	144.65	Vitamin B-12 -Case (Deficiency Vitamin B12 Deficiency)	QATARI	MALE	44
**101**	159.75	142	162	109.22	Vitamin B-12 -Case (Deficiency Vitamin B12 Deficiency)	QATARI	FEMALE	27
**102**	158.72	136	140	135.79	Vitamin B-12 -Case (Deficiency Vitamin B12 Deficiency)	QATARI	FEMALE	44
**103**	116.54	124	130	76.75	Vitamin B-12 -Case (Deficiency Vitamin B12 Deficiency)	ARAB	FEMALE	25
**104**	131.60	142	149	246.49	Vitamin B-12 -Case (Deficiency Vitamin B12 Deficiency)	QATARI	MALE	25
**105**	176.98	122	131	127.67	Vitamin B-12 -Case (Deficiency Vitamin B12 Deficiency)	QATARI	FEMALE	44
**106**	182.44	130	142	91.51	Vitamin B-12 -Case (Deficiency Vitamin B12 Deficiency)	QATARI	FEMALE	31
**107**	126.32	130	142	113.65	Vitamin B-12 -Case (Deficiency Vitamin B12 Deficiency)	QATARI	FEMALE	42
**108**	146.35	134	139	127.67	Vitamin B-12 -Case (Deficiency Vitamin B12 Deficiency)	ARAB	FEMALE	60
**109**	88.49	<111	120	158.67	Vitamin B-12 -Case (Deficiency Vitamin B12 Outliers)	QATARI	MALE	52
**110**	301.42	229	240	145.39	Vitamin B-12 -Control (Normal Vitamin B12)	QATARI	FEMALE	53
**111**	122.57	116	140	126.20	Vitamin B-12 -Case (Deficiency Vitamin B12 Deficiency)	QATARI	FEMALE	38
**112**	117.15	<111	124	393.35	Vitamin B-12 -Case (Deficiency Vitamin B12 Outliers)	QATARI	FEMALE	38
**113**	186.78	130	157	100.37	Vitamin B-12 -Case (Deficiency Vitamin B12 Deficiency)	QATARI	FEMALE	38
**114**	192.18	131	161	114.39	Vitamin B-12 -Case (Deficiency Vitamin B12 Deficiency)	QATARI	FEMALE	21
**115**	228.77	128	151	96.68	Vitamin B-12 -Case (Deficiency Vitamin B12 Deficiency)	QATARI	FEMALE	20
**116**	168.03	147	181	113.65	Vitamin B-12 -Case (Deficiency Vitamin B12 Deficiency)	QATARI	FEMALE	51
**117**	152.07	118	146	126.94	Vitamin B-12 -Case (Deficiency Vitamin B12 Deficiency)	ARAB	FEMALE	40

**118**	595.40	443	457	276.75	Vitamin B-12 -Control (Normal Vitamin B12)	QATARI	MALE	43
**119**	134.57	<111	126	333.58	Vitamin B-12 -Case (Deficiency Vitamin B12 Outliers)	QATARI	FEMALE	26
**120**	147.18	135	146	152.77	Vitamin B-12 -Case (Deficiency Vitamin B12 Deficiency)	QATARI	FEMALE	25
**121**	143.31	<111	138	112.18	Vitamin B-12 -Case (Deficiency Vitamin B12 Outliers)	QATARI	FEMALE	54
**122**	164.78	118	132	114.39	Vitamin B-12 -Case (Deficiency Vitamin B12 Deficiency)	QATARI	FEMALE	34
**123**	189.45	135	158	102.58	Vitamin B-12 -Case (Deficiency Vitamin B12 Deficiency)	QATARI	FEMALE	38
**124**	396.23	280	310	1443.53	Vitamin B-12 -Control (Normal Vitamin B12)	ARAB	MALE	43
**125**	552.72	355	375	1470.10	Vitamin B-12 -Control (Normal Vitamin B12)	QATARI	FEMALE	23
**126**	192.19	141	177	123.25	Vitamin B-12 -Case (Deficiency Vitamin B12 Deficiency)	QATARI	FEMALE	38
**127**	151.30	<111	125	117.34	Vitamin B-12 -Case (Deficiency Vitamin B12 Outliers)	QATARI	MALE	46
**128**	158.71	<111	143	63.47	Vitamin B-12 -Case (Deficiency Vitamin B12 Outliers)	QATARI	FEMALE	36
**129**	146.10	128	191	142.43	Vitamin B-12 -Case (Deficiency Vitamin B12 Deficiency)	QATARI	FEMALE	37
**130**	1405.23	>1476	1771	135.79	Vitamin B-12 -Case (Deficiency Vitamin B12 Outliers)	QATARI	FEMALE	61
**131**	1333.57	>1476	1651	125.46	Vitamin B-12 -Case (Deficiency Vitamin B12 Outliers)	QATARI	FEMALE	55
**132**	160.63	<111	144	104.80	Vitamin B-12 -Case (Deficiency Vitamin B12 Outliers)	QATARI	FEMALE	51
**133**	191.19	124	146	139.48	Vitamin B-12 -Case (Deficiency Vitamin B12 Deficiency)	QATARI	FEMALE	32
**134**	82.18	<111	107	107.75	Vitamin B-12 -Case (Deficiency Vitamin B12 Outliers)	QATARI	MALE	38
**135**	204.16	133	150	123.25	Vitamin B-12 -Case (Deficiency Vitamin B12 Deficiency)	QATARI	FEMALE	43
**136**	438.84	341	367	142.43	Vitamin B-12 -Control (Normal Vitamin B12)	QATARI	FEMALE	25
**137**	145.41	118	139	113.65	Vitamin B-12 -Case (Deficiency Vitamin B12 Deficiency)	QATARI	MALE	24
**138**	131.64	140	144	131.36	Vitamin B-12 -Case (Deficiency Vitamin B12 Deficiency)	QATARI	FEMALE	44
**139**	171.92	142	147	98.89	Vitamin B-12 -Case (Deficiency Vitamin B12 Deficiency)	QATARI	FEMALE	32

**140**	160.13	135	156	118.82	Vitamin B-12 -Case (Deficiency Vitamin B12 Deficiency)	QATARI	FEMALE	30
**141**	125.66	126	131	133.58	Vitamin B-12 -Case (Deficiency Vitamin B12 Deficiency)	QATARI	MALE	44
**142**	177.53	133	150	140.22	Vitamin B-12 -Case (Deficiency Vitamin B12 Deficiency)	QATARI	FEMALE	57
**143**	145.12	146	150	173.43	Vitamin B-12 -Case (Deficiency Vitamin B12 Deficiency)	QATARI	FEMALE	23
**144**	1515.70	>1476	1754	156.46	Vitamin B-12 -Case (Deficiency Vitamin B12 Outliers)	QATARI	FEMALE	52
**145**	189.41	128	137	134.32	Vitamin B-12 -Case (Deficiency Vitamin B12 Deficiency)	QATARI	FEMALE	30
**146**	181.01	121	146	1453.86	Vitamin B-12 -Case (Deficiency Vitamin B12 Deficiency)	QATARI	FEMALE	33
**147**	159.17	120	152	154.24	Vitamin B-12 -Case (Deficiency Vitamin B12 Deficiency)	QATARI	FEMALE	61
**148**	166.32	141	163	292.99	Vitamin B-12 -Case (Deficiency Vitamin B12 Deficiency)	QATARI	MALE	30
**149**	168.40	<111	130	133.58	Vitamin B-12 -Case (Deficiency Vitamin B12 Outliers)	ARAB	FEMALE	34
**150**	196.56	141	166	126.94	Vitamin B-12 -Case (Deficiency Vitamin B12 Deficiency)	QATARI	FEMALE	44
**151**	178.62	126	145	169.74	Vitamin B-12 -Case (Deficiency Vitamin B12 Deficiency)	QATARI	FEMALE	38
**152**	134.46	126	133	129.15	Vitamin B-12 -Case (Deficiency Vitamin B12 Deficiency)	QATARI	FEMALE	46
**153**	1331.20	>1476	1581	351.29	Vitamin B-12 -Case (Deficiency Vitamin B12 Outliers)	QATARI	FEMALE	43
**154**	131.19	<111	130	315.13	Vitamin B-12 -Case (Deficiency Vitamin B12 Outliers)	QATARI	MALE	26
**155**	361.18	285	310	240.59	Vitamin B-12 -Control (Normal Vitamin B12)	QATARI	FEMALE	39
**156**	136.35	<111	135	138.74	Vitamin B-12 -Case (Deficiency Vitamin B12 Outliers)	QATARI	FEMALE	40
**157**	107.05	<111	119	120.29	Vitamin B-12 -Case (Deficiency Vitamin B12 Outliers)	ARAB	FEMALE	32
**158**	133.49	<111	118	239.85	Vitamin B-12 -Case (Deficiency Vitamin B12 Outliers)	QATARI	FEMALE	41
**159**	116.94	<111	120	99.63	Vitamin B-12 -Case (Deficiency Vitamin B12 Outliers)	QATARI	FEMALE	29
**160**	478.22	N/A	351	67.90	Vitamin B-12 -Case (Deficiency Vitamin B12 Outliers)	QATARI	FEMALE	48
**161**	408.73	300	321	144.65	Vitamin B-12 -Control (Normal Vitamin B12)	QATARI	FEMALE	38

**162**	315.52	231	251	109.22	Vitamin B-12 -Control (Normal Vitamin B12)	QATARI	FEMALE	54
**163**	135.82	<111	133	123.25	Vitamin B-12 -Case (Deficiency Vitamin B12 Outliers)	QATARI	FEMALE	39
**164**	92.43	<111	116	285.61	Vitamin B-12 -Case (Deficiency Vitamin B12 Outliers)	QATARI	FEMALE	40
**165**	284.06	226	259	124.72	Vitamin B-12 -Control (Normal Vitamin B12)	QATARI	FEMALE	43
**166**	80.75	<111	116	124.72	Vitamin B-12 -Case (Deficiency Vitamin B12 Outliers)	ARAB	MALE	45
**167R**	48.46	<111	114	98.89	Vitamin B-12 -Case (Deficiency Vitamin B12 Outliers)	QATARI	MALE	51
**168**	1437.55	>1476	1660	122.51	Vitamin B-12 -Case (Deficiency Vitamin B12 Outliers)	QATARI	FEMALE	57
**169**	142.09	<111	120	105.53	Vitamin B-12 -Case (Deficiency Vitamin B12 Outliers)	QATARI	FEMALE	33
**170**	99.41	<111	124	362.36	Vitamin B-12 -Case (Deficiency Vitamin B12 Outliers)	QATARI	FEMALE	37
**171**	87.25	<111	136	100.37	Vitamin B-12 -Case (Deficiency Vitamin B12 Outliers)	QATARI	MALE	33
**172**	389.22	257	281	107.75	Vitamin B-12 -Control (Normal Vitamin B12)	ARAB	FEMALE	48
**173**	146.90	<111	111	94.46	Vitamin B-12 -Case (Deficiency Vitamin B12 Outliers)	ARAB	FEMALE	52
**174**	140.72	<111	109	104.80	Vitamin B-12 -Case (Deficiency Vitamin B12 Outliers)	ARAB	FEMALE	56
**175**	116.10	<111	131	112.18	Vitamin B-12 -Case (Deficiency Vitamin B12 Outliers)	ARAB	MALE	58
**176**	128.15	<111	135	114.39	Vitamin B-12 -Case (Deficiency Vitamin B12 Outliers)	ARAB	MALE	21
**177**	1458.29	>1476	1690	112.91	Vitamin B-12 -Case (Deficiency Vitamin B12 Outliers)	QATARI	FEMALE	56
**178**	125.25	<111	119	239.11	Vitamin B-12 -Case (Deficiency Vitamin B12 Outliers)	ARAB	FEMALE	21
**179**	413.24	335	342	539.48	Vitamin B-12 -Control (Normal Vitamin B12)	ARAB	FEMALE	28
**180**	127.89	<111	129	348.34	Vitamin B-12 -Case (Deficiency Vitamin B12 Outliers)	QATARI	FEMALE	60
**181**	111.04	<111	115	143.17	Vitamin B-12 -Case (Deficiency Vitamin B12 Outliers)	ARAB	FEMALE	37
**182**	1543.75	>1476	1804	122.51	Vitamin B-12 -Case (Deficiency Vitamin B12 Outliers)	QATARI	FEMALE	46
**183**	113.10	<111	120	107.01	Vitamin B-12 -Case (Deficiency Vitamin B12 Outliers)	ARAB	FEMALE	36
**184**	145.85	<111	136	363.10	Vitamin B-12 -Case (Deficiency Vitamin B12 Outliers)	QATARI	FEMALE	38
**185**	81.93	<111	116	281.92	Vitamin B-12 -Case (Deficiency Vitamin B12 Outliers)	QATARI	FEMALE	40
**186**	124.72	<111	123	253.13	Vitamin B-12 -Case (Deficiency Vitamin B12 Outliers)	QATARI	MALE	60
**187**	147.63	<111	117	261.25	Vitamin B-12 -Case (Deficiency Vitamin B12 Outliers)	QATARI	FEMALE	60
**188**	290.02	242	251	363.83	Vitamin B-12 -Control (Normal Vitamin B12)	QATARI	MALE	41
**189**	778.96	541	560	259.04	Vitamin B-12 -Control (Normal Vitamin B12)	QATARI	MALE	67
**190**	430.69	345	361	422.14	Vitamin B-12 -Control (Normal Vitamin B12)	QATARI	FEMALE	36
**191**	172.51	<111	119	253.13	Vitamin B-12 -Case (Deficiency Vitamin B12 Outliers)	QATARI	MALE	24
**192**	138.23	<111	145	292.25	Vitamin B-12 -Case (Deficiency Vitamin B12 Outliers)	QATARI	MALE	37

**193**	120.01	<111	119	300.37	Vitamin B-12 -Case (Deficiency Vitamin B12 Outliers)	QATARI	FEMALE	59
**194**	532.15	360	376	324.72	Vitamin B-12 -Control (Normal Vitamin B12)	ARAB	MALE	22
**195**	363.24	235	246	295.20	Vitamin B-12 -Control (Normal Vitamin B12)	QATARI	MALE	28
**196**	327.66	243	267	352.03	Vitamin B-12 -Control (Normal Vitamin B12)	QATARI	MALE	32
**197**	312.49	244	261	351.29	Vitamin B-12 -Control (Normal Vitamin B12)	QATARI	FEMALE	32
**198**	436.91	321	335	348.34	Vitamin B-12 -Control (Normal Vitamin B12)	QATARI	MALE	35
**199**	337.37	231	341	290.77	Vitamin B-12 -Control (Normal Vitamin B12)	QATARI	MALE	47
**200**	608.02	382	396	342.43	Vitamin B-12 -Control (Normal Vitamin B12)	QATARI	MALE	41
**201**	380.08	250	288	332.84	Vitamin B-12 -Control (Normal Vitamin B12)	QATARI	MALE	40
**202**	390.29	225	250	440.59	Vitamin B-12 -Control (Normal Vitamin B12)	QATARI	FEMALE	21
**203**	402.93	285	293	551.29	Vitamin B-12 -Control (Normal Vitamin B12)	QATARI	MALE	48
**204**	365.89	252	291	292.99	Vitamin B-12 -Control (Normal Vitamin B12)	QATARI	MALE	42
**205**	516.89	311	340	261.25	Vitamin B-12 -Control (Normal Vitamin B12)	QATARI	MALE	49
**206**	395.09	228	256	351.29	Vitamin B-12 -Control (Normal Vitamin B12)	QATARI	MALE	30
**207**	494.39	320	346	388.93	Vitamin B-12 -Control (Normal Vitamin B12)	QATARI	FEMALE	39
**208**	453.39	276	315	429.52	Vitamin B-12 -Control (Normal Vitamin B12)	QATARI	FEMALE	21
**209**	452.62	300	316	282.65	Vitamin B-12 -Control (Normal Vitamin B12)	QATARI	FEMALE	70
**210**	446.55	247	291	355.72	Vitamin B-12 -Control (Normal Vitamin B12)	QATARI	MALE	36
**211**	447.50	294	306	223.61	Vitamin B-12 -Control (Normal Vitamin B12)	QATARI	MALE	48
**212**	458.67	303	330	281.18	Vitamin B-12 -Control (Normal Vitamin B12)	QATARI	FEMALE	32
**213**	621.92	383	397	456.08	Vitamin B-12 -Control (Normal Vitamin B12)	QATARI	FEMALE	33
**214**	990.84	524	551	202.21	Vitamin B-12 -Control (Normal Vitamin B12)	QATARI	MALE	28
**215**	346.62	296	310	353.50	Vitamin B-12 -Control (Normal Vitamin B12)	QATARI	FEMALE	32
**216**	334.43	253	261	374.90	Vitamin B-12 -Control (Normal Vitamin B12)	QATARI	MALE	50
**217**	435.86	354	362	215.50	Vitamin B-12 -Control (Normal Vitamin B12)	QATARI	MALE	36
**218**	553.34	394	410	238.37	Vitamin B-12 -Control (Normal Vitamin B12)	QATARI	FEMALE	55
**219**	546.82	396	406	334.31	Vitamin B-12 -Control (Normal Vitamin B12)	QATARI	MALE	45
**220**	390.95	332	331	251.66	Vitamin B-12 -Control (Normal Vitamin B12)	QATARI	MALE	50
**221**	527.88	440	441	302.58	Vitamin B-12 -Control (Normal Vitamin B12)	ARAB	FEMALE	38
**222**	317.47	252	257	215.50	Vitamin B-12 -Control (Normal Vitamin B12)	QATARI	FEMALE	50
**223**	381.41	348	342	408.85	Vitamin B-12 -Control (Normal Vitamin B12)	QATARI	FEMALE	32

**224**	787.15	561	546	271.58	Vitamin B-12 -Control (Normal Vitamin B12)	ARAB	FEMALE	69
**225**	296.36	252	255	348.34	Vitamin B-12 -Control (Normal Vitamin B12)	QATARI	FEMALE	54
**226**	518.16	402	403	247.97	Vitamin B-12 -Control (Normal Vitamin B12)	QATARI	FEMALE	32
**227**	647.31	459	466	182.29	Vitamin B-12 -Control (Normal Vitamin B12)	ARAB	MALE	54
**228**	267.38	241	246	594.09	Vitamin B-12 -Control (Normal Vitamin B12)	QATARI	FEMALE	32
**229**	312.44	293	297	372.69	Vitamin B-12 -Control (Normal Vitamin B12)	ARAB	MALE	38
**230**	535.06	376	379	292.25	Vitamin B-12 -Control (Normal Vitamin B12)	ARAB	MALE	27
**231**	372.18	262	271	180.81	Vitamin B-12 -Control (Normal Vitamin B12)	QATARI	MALE	66
**232**	454.03	325	337	195.57	Vitamin B-12 -Control (Normal Vitamin B12)	ARAB	MALE	40
**233**	315.66	251	255	205.16	Vitamin B-12 -Control (Normal Vitamin B12)	QATARI	MALE	34
**234**	577.47	481	486	292.99	Vitamin B-12 -Control (Normal Vitamin B12)	QATARI	MALE	45
**235**	346.59	299	301	341.69	Vitamin B-12 -Control (Normal Vitamin B12)	QATARI	FEMALE	45
**236**	496.58	406	411	245.02	Vitamin B-12 -Control (Normal Vitamin B12)	QATARI	MALE	27
**237**	359.02	311	319	436.90	Vitamin B-12 -Control (Normal Vitamin B12)	ARAB	MALE	41
**238**	240.14	222	230	309.96	Vitamin B-12 -Control (Normal Vitamin B12)	QATARI	FEMALE	48
**239**	925.01	729	730	233.21	Vitamin B-12 -Control (Normal Vitamin B12)	ARAB	MALE	63
**240**	577.65	447	450	299.63	Vitamin B-12 -Control (Normal Vitamin B12)	QATARI	MALE	63
**241**	410.84	351	353	321.03	Vitamin B-12 -Control (Normal Vitamin B12)	OTHER	MALE	42
**242**	234.57	224	227	265.68	Vitamin B-12 -Control (Normal Vitamin B12)	OTHER	FEMALE	29
**243**	267.93	229	233	644.27	Vitamin B-12 -Control (Normal Vitamin B12)	QATARI	FEMALE	19
**244**	255.64	231	236	331.36	Vitamin B-12 -Control (Normal Vitamin B12)	ARAB	FEMALE	53
**245**	394.16	333	337	271.58	Vitamin B-12 -Control (Normal Vitamin B12)	QATARI	FEMALE	29
**246**	506.36	381	390	194.83	Vitamin B-12 -Control (Normal Vitamin B12)	QATARI	MALE	66
**247**	382.79	283	291	273.06	Vitamin B-12 -Control (Normal Vitamin B12)	ARAB	FEMALE	28
**248**	621.59	516	514	248.71	Vitamin B-12 -Control (Normal Vitamin B12)	QATARI	MALE	35
**249**	418.64	385	382	331.36	Vitamin B-12 -Control (Normal Vitamin B12)	QATARI	MALE	28
**250**	270.54	242	266	280.44	Vitamin B-12 -Control (Normal Vitamin B12)	ARAB	MALE	34
**251**	406.28	349	351	395.57	Vitamin B-12 -Control (Normal Vitamin B12)	QATARI	MALE	26
**252**	519.12	406	410	309.96	Vitamin B-12 -Control (Normal Vitamin B12)	QATARI	FEMALE	61
**253**	466.89	306	321	351.29	Vitamin B-12 -Control (Normal Vitamin B12)	QATARI	MALE	35
**254**	986.71	694	702	322.51	Vitamin B-12 -Control (Normal Vitamin B12)	QATARI	MALE	29

**255**	503.80	387	391	356.45	Vitamin B-12 -Control (Normal Vitamin B12)	QATARI	MALE	23
**256**	435.27	337	340	298.15	Vitamin B-12 -Control (Normal Vitamin B12)	QATARI	MALE	32
**257**	271.50	225	230	412.54	Vitamin B-12 -Control (Normal Vitamin B12)	QATARI	MALE	35
**258**	374.28	307	310	154.98	Vitamin B-12 -Control (Normal Vitamin B12)	ARAB	MALE	62
**259**	414.00	310	336	436.90	Vitamin B-12 -Control (Normal Vitamin B12)	OTHER	MALE	36
**260**	491.42	402	401	360.14	Vitamin B-12 -Control (Normal Vitamin B12)	QATARI	FEMALE	40
**261**	424.88	336	340	435.42	Vitamin B-12 -Control (Normal Vitamin B12)	QATARI	FEMALE	24
**262**	658.22	493	492	180.07	Vitamin B-12 -Control (Normal Vitamin B12)	QATARI	MALE	35
**263**	397.63	340	340	211.81	Vitamin B-12 -Control (Normal Vitamin B12)	ARAB	FEMALE	58
**264**	502.47	428	430	456.08	Vitamin B-12 -Control (Normal Vitamin B12)	QATARI	MALE	42
**265**	442.19	337	343	368.26	Vitamin B-12 -Control (Normal Vitamin B12)	ARAB	MALE	67
**266**	429.16	359	361	474.53	Vitamin B-12 -Control (Normal Vitamin B12)	ARAB	MALE	25
**267**	487.01	363	371	541.69	Vitamin B-12 -Control (Normal Vitamin B12)	OTHER	MALE	26
**268**	605.49	406	430	374.90	Vitamin B-12 -Control (Normal Vitamin B12)	ARAB	MALE	38
**269**	208.28	222	226	234.68	Vitamin B-12 -Control (Normal Vitamin B12)	QATARI	MALE	47
**270**	686.41	520	526	380.81	Vitamin B-12 -Control (Normal Vitamin B12)	QATARI	FEMALE	35
**271**	555.50	426	430	338.00	Vitamin B-12 -Control (Normal Vitamin B12)	ARAB	FEMALE	49
**272**	632.72	473	483	302.58	Vitamin B-12 -Control (Normal Vitamin B12)	QATARI	FEMALE	51
**273**	275.74	245	251	352.03	Vitamin B-12 -Control (Normal Vitamin B12)	QATARI	MALE	50
**274**	316.12	240	250	430.99	Vitamin B-12 -Control (Normal Vitamin B12)	QATARI	MALE	44
**275**	773.42	545	567	193.36	Vitamin B-12 -Control (Normal Vitamin B12)	ARAB	FEMALE	40
**276**	564.89	464	471	186.71	Vitamin B-12 -Control (Normal Vitamin B12)	QATARI	FEMALE	47
**277**	670.56	533	540	315.13	Vitamin B-12 -Control (Normal Vitamin B12)	ARAB	MALE	40
**278**	923.16	678	681	290.77	Vitamin B-12 -Control (Normal Vitamin B12)	QATARI	MALE	29
**279**	507.58	405	410	305.53	Vitamin B-12 -Control (Normal Vitamin B12)	QATARI	MALE	21
**280**	299.35	271	282	224.35	Vitamin B-12 -Control (Normal Vitamin B12)	ARAB	FEMALE	33
**281**	546.83	411	422	281.92	Vitamin B-12 -Control (Normal Vitamin B12)	QATARI	MALE	49
**282**	457.04	342	355	202.21	Vitamin B-12 -Control (Normal Vitamin B12)	QATARI	MALE	46
**283**	490.29	311	340	395.57	Vitamin B-12 -Control (Normal Vitamin B12)	ARAB	MALE	43
**284**	626.80	405	430	267.16	Vitamin B-12 -Control (Normal Vitamin B12)	QATARI	MALE	43
**285**	533.68	496	500	374.90	Vitamin B-12 -Control (Normal Vitamin B12)	ARAB	FEMALE	36

**286**	305.09	233	252	465.68	Vitamin B-12 -Control (Normal Vitamin B12)	QATARI	MALE	33
**287**	298.71	224	240	253	Vitamin B-12 -Control (Normal Vitamin B12)	QATARI	FEMALE	48
**288**	482.72	351	364	427	Vitamin B-12 -Control (Normal Vitamin B12)	QATARI	FEMALE	35
**289**	460.48	403	406	394	Vitamin B-12 -Control (Normal Vitamin B12)	QATARI	MALE	26
**290**	474.37	351	360	414	Vitamin B-12 -Control (Normal Vitamin B12)	QATARI	MALE	73
**291**	322.52	299	301	304	Vitamin B-12 -Control (Normal Vitamin B12)	ARAB	MALE	28
**292**	408.28	344	349	382	Vitamin B-12 -Control (Normal Vitamin B12)	ARAB	MALE	36
**293**	266.91	223	246	274	Vitamin B-12 -Control (Normal Vitamin B12)	ARAB	FEMALE	35
**294**	607.73	461	466	536	Vitamin B-12 -Control (Normal Vitamin B12)	QATARI	MALE	23
**295**	419.95	321	330	362	Vitamin B-12 -Control (Normal Vitamin B12)	QATARI	FEMALE	23
**296**	590.78	438	459	508	Vitamin B-12 -Control (Normal Vitamin B12)	QATARI	FEMALE	19
**297**	711.45	513	536	631	Vitamin B-12 -Control (Normal Vitamin B12)	QATARI	MALE	29
